# Multiple sclerosis-associated HLA demarcates EBV-specific CD8^+^ T cells with an exhausted and brain residency phenotype

**DOI:** 10.1016/j.isci.2026.115744

**Published:** 2026-04-15

**Authors:** Sanne Reijm, Ana M. Marques, Jasper Rip, Cato E.A. Corsten, Annet F. Wierenga-Wolf, Harm de Wit, Marie-José Melief, Yifan van Hasselt, Jamie van Langelaar, Rinze Neuteboom, Beatrijs H.A. Wokke, Yvonne M. Mueller, Joost Smolders, Marvin M. van Luijn

**Affiliations:** 1 Department of Immunology, Erasmus MC, University Medical Center Rotterdam, Rotterdam, the Netherlands; 2 MS Center ErasMS, Erasmus MC, University Medical Center Rotterdam, Rotterdam, the Netherlands; 3 Department of Neurology, Erasmus MC, University Medical Center Rotterdam, Rotterdam, the Netherlands; 4 Neuroimmunology Research Group, Netherlands Institute for Neuroscience, Amsterdam, the Netherlands

**Keywords:** immunology

## Abstract

In multiple sclerosis (MS), T cells could contribute to disease in their attempt to control the Epstein-Barr virus (EBV). Here, we compared the presence of HLA-B7 (higher MS risk) or HLA-A2 (lower MS risk) and investigated the effector phenotype of EBV epitope-specific CD8^+^ T cells in MS using spectral flow cytometry. In contrast to HLA-A2, HLA-B7-restricted CD8^+^ T cells recognized few EBV epitopes. These HLA-B7-restricted EBV-specific CD8^+^ T cells expressed CNS residency markers and were most abundant in postmortem CNS compartments of an HLA-A2^+^B7^+^ MS donor. HLA-B7-restricted EBV-specific CD8^+^ T cells displayed a more exhausted phenotype (PD-1^+^CD244^+^CD160^+^KLRG1^+^TIGIT^+^). In line with these findings, anti-EBNA1 IgG levels were elevated in patients with MS carrying HLA-B7 but lacking HLA-A2. These data support a model in which the confined response against EBV generates circulating HLA-B7-restricted CD8^+^ T cells less able to control EBV and more prone to infiltrate the CNS.

## Highlights

• CD20 and CXCR3 are higher expressed by EBV- than CMV-specific CD8^+^ T cells
• HLA-B7-restricted EBV-specific CD8^+^ T cells mainly recognize EBNA3A
• EBV-specific CD8^+^ T cells restricted to HLA-B7 display an exhausted phenotype
• HLA-B7 and HLA-A2 carriership is associated with different anti-EBNA1 IgG levels in MS

## Graphical abstract

**Figure:**
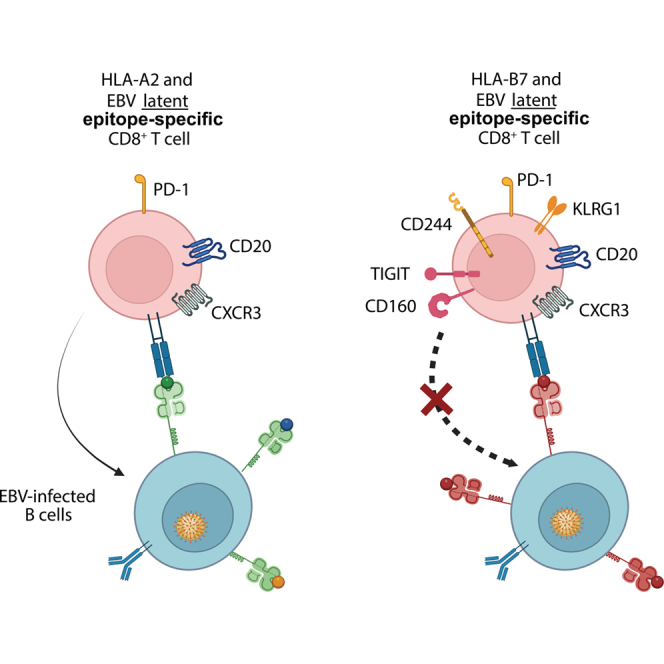

## Introduction

Multiple sclerosis (MS) is a chronic inflammatory disease leading to neurodegeneration in the central nervous system (CNS). The cause of MS is unknown, but genetic predisposition together with environmental influences are considered to be the disease trigger. The major environmental susceptibility factor for MS is an infection with the Epstein-Barr virus (EBV), as now proven by a required antibody response prior to onset. An infection with the cytomegalovirus (CMV), another persistent herpesvirus, does not increase but might even decrease this risk.^1^ Furthermore, it has been shown that antibody levels against EBV are increased in patients with MS (pwMSs) compared to the healthy population.^2^ Genetic predisposition has been shown by large genome-wide association studies. Here, 233 genetic risk variants were identified, of which 32 are located in the HLA region.^3^ This region is the by far the strongest associated with MS susceptibility and the presence of certain HLA alleles can have a risk or protective effect on MS onset.^4^^,^^5^

Immune infiltration in the brain is a hallmark of MS attacks. While B cells can infiltrate the CNS in pwMS, these cells are rarely present in a healthy CNS.^6^ B cells are the target cells for EBV and CD8^+^ T cells are important players in controlling viral infections. Since CD8^+^ T cells can recognize EBV-derived epitopes in MS-associated HLA class I alleles presented by infected B cells, these cells might be involved in the aberrant immune response against EBV in MS. CD8^+^ T cells are indeed described as the major fraction of T cells populating the brain of pwMS,^7^ aberrant recruitment of CD8^+^ T cells has been described in MS cerebrospinal fluid (CSF),^8^ and CD8^+^ T cells directed against EBV antigens have been identified in the CSF of pwMS.^9^ The strongest protective and risk HLA alleles for MS susceptibility are HLA-A2 and HLA-DRB1∗15:01, respectively.^4^ Since HLA class I molecule HLA-B7 is in strong linkage disequilibrium with HLA-DRB1∗15:01, HLA-A2 and HLA-B7 were included in this study to investigate the EBV-specific CD8^+^ T cell response.

Lower expression of pro-inflammatory cytokines and cytolytic proteins by EBV-specific CD8^+^ T cells after antigen stimulation has been described for the HLA-B7^+^ pwMS compared to healthy donors.^10^ Moreover, EBV viral load was found to be higher in HLA-B7^+^ compared to HLA-A2^+^ pwMS.^11^ Together, this suggests that HLA-B7 and/or HLA-A2 carriage determines the ability of CD8^+^ T cells to control the immune response against EBV inside and/or outside the CNS in persons who develop MS. However, little is known about how epitope-specificities to EBV distinctively shape the effector phenotype of CD8^+^ T cells and their potential to infiltrate the CNS in the context of protective and risk HLA alleles. Together with a potential role in EBV seroprevalence, deeper analysis of such T cell subsets could improve the understanding of MS susceptibility and support the development of EBV-specific T cell therapies. Therefore, we performed an in-depth phenotypical characterization of HLA-B7- and HLA-A2-restricted CD8^+^ T cells specific for different EBV and CMV epitopes from the blood of healthy individuals and treatment-naive as well as natalizumab-treated pwMS. The treatment with natalizumab (anti-VLA-4) allowed us to analyze CNS-homing CD8^+^ T cells that are trapped in the blood. Furthermore, we assessed the recruitment and effector phenotype of epitope-specific CD8^+^ T cells in different postmortem CNS compartments from an MS donor *ex vivo*.

## Results

### The number of EBV epitopes is confined for HLA-B7-restricted CD8^+^ T cells

**Figure 1:**
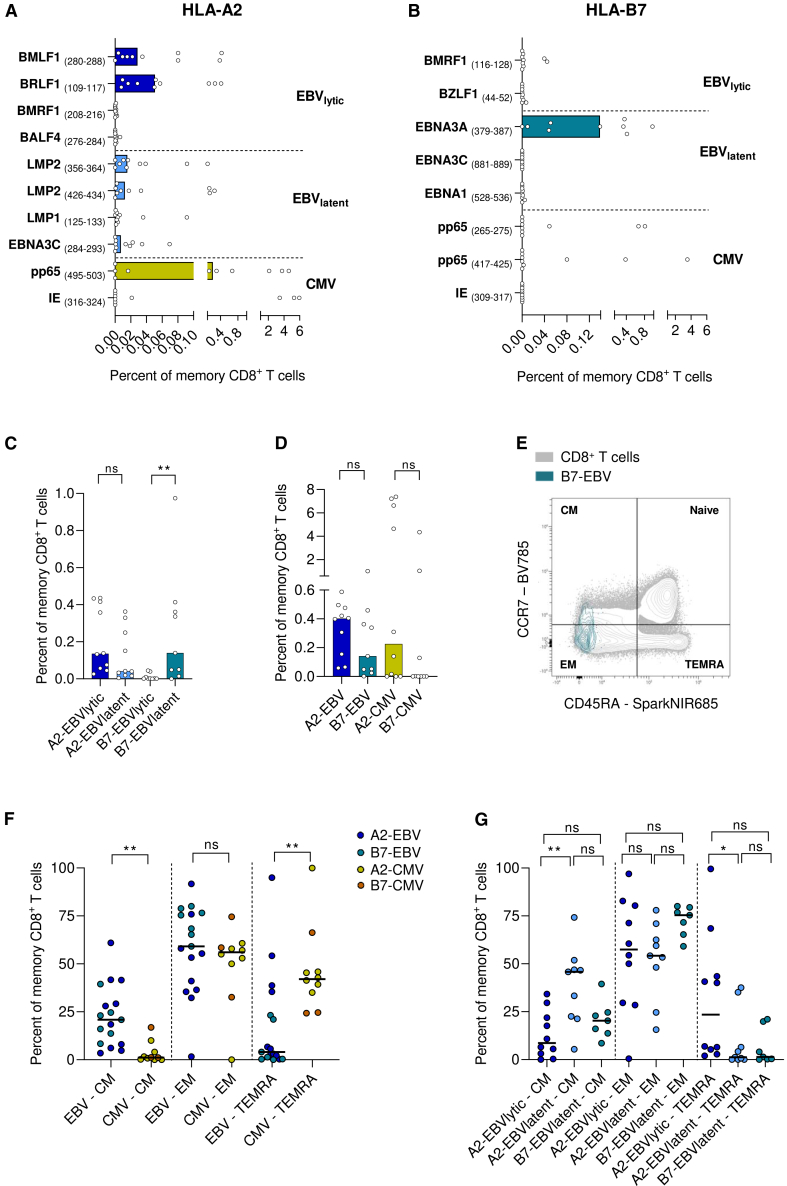
Frequencies and differentiation of EBV- and CMV-specific CD8^+^ T cells in HD Percentage of EBV- and CMV-specific CD8^+^ T cells of total memory CD8^+^ T cells per immunodominant epitope restricted to HLA-A2 (A) or HLA-B7 (B). (C) Percentages of EBV-specific CD8^+^ T cells of total memory CD8^+^ T cells restricted to latent or lytic peptides. (D) Percentage of total EBV- and CMV-specific CD8^+^ T cells of total memory CD8^+^ T cells. (E) Representative example of the gating strategy of the differentiation states. (F) Percentage of total EBV- and CMV-specific CD8^+^ T cells of total memory CD8^+^ T cells per differentiation state. (G) Percentages of EBV-specific CD8^+^ T cells of total memory CD8^+^ T cells restricted to latent or lytic peptides per differentiation state. Mann-Whitney test (C, D, and F), or Kruskal-Wallis test with Dunn posthoc (G) was used for statistical testing. Every dot represents one individual. Bars (A–D) or lines (F–G) represent medians. ∗*p* < 0.05, ∗∗*p* < 0.01, ns = not significant. See also Figures S1 and S2.

We first investigated the effect of HLA-B7 and HLA-A2 on the presence and epitope-specificities of EBV-specific CD8^+^ T cells in the blood of healthy donors (HDs). CMV-specific CD8^+^ T cells served as antigen-specific controls. Antigen-specific cells were detected by a combinatorial staining method with HLA class I tetramers (Figure S1A), resulting in simultaneous detection of CD8^+^ T cells recognizing ten different HLA-A2-restricted or eight different HLA-B7-restricted EBV- and CMV immunodominant epitopes. A representative staining of HLA-A2- and HLA-B7 restricted CD8^+^ T cells from HD blood is shown in Figures S1B and S1C, respectively. We determined the frequencies of EBV- and CMV-specific CD8^+^ T cells specific for immunodominant epitopes presented in HLA-A2 or HLA-B7. For EBV, epitopes from both lytic and latent proteins were included. For HLA-A2, various lytic and latent epitopes were recognized by the EBV-specific CD8^+^ T cells (Figure 1A). However, for HLA-B7, the response was restricted to mainly one latent epitope, namely EBNA3A (379–387) (Figure 1B). For subsequent analysis, CD8^+^ T cells were distinguished based their specificity to EBV latent or lytic epitopes. More EBV latent than lytic epitopes were recognized by HLA-B7-restricted CD8^+^ T cells, which was not the case for EBV-specific CD8^+^ T cells restricted to HLA-A2 (Figure 1C). We found no significant differences in total EBV- or CMV-specific CD8^+^ T cell frequencies between HLA-A2 and HLA-B7 carriers (Figure 1D).

### Antigen-specificity and HLA distinctively shapes the EBV-specific CD8^+^ T cell memory state

Next, we investigated how the presence of HLA-B7 and HLA-A2 is associated with the memory state of EBV- and CMV-specific CD8^+^ T cells based on the expression of CCR7 and CD45RA (Figure 1E). The full gating strategy of a representative donor is shown in Figure S2A. CMV-specific CD8^+^ T cells especially showed an effector memory (EM) or terminally differentiated EM (TEMRA) phenotype, while EBV-specific CD8^+^ T cells were also found within the central memory (CM) compartment (Figure 1F). Within the pool of HLA-A2-restricted EBV-specific CD8^+^ T cells, those specific for EBV lytic epitopes were mostly EM and TEMRA and those specific for EBV latent epitopes were mostly CM (Figure 1G). This could be explained by the nature of the epitopes, as lytic proteins are expressed during the replication phase where immediate effector functions are needed and T_CM_ cells are primary involved in long-term surveillance during the latent phase of an EBV infection. However, HLA-B7-restricted CD8^+^ T cells specific for EBV latent epitopes displayed a memory phenotype that was similar to HLA-A2-restricted CD8^+^ T cells specific for EBV lytic epitopes and tended to display an EM phenotype (Figure 1G).

### Natalizumab restores the decrease of EBV-specific CD8^+^ T_EM_ cells in pwMS

**Figure 2:**
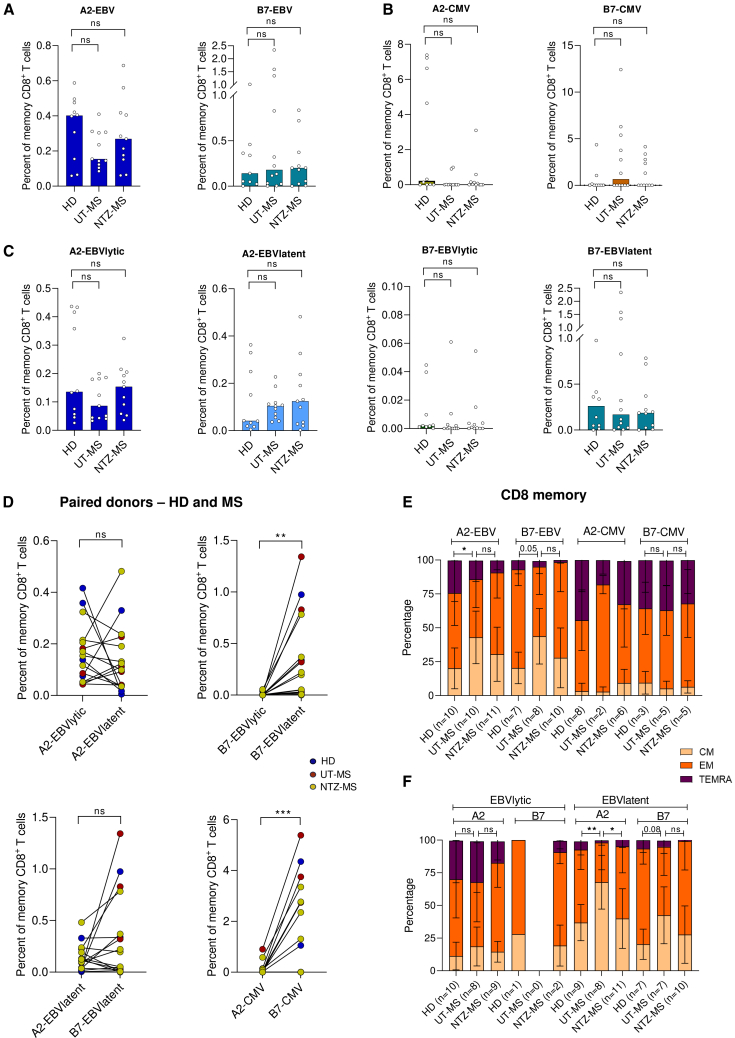
Frequencies and differentiation of EBV- and CMV-specific CD8^+^ T cells in MS Percentage of antigen-specific CD8^+^ T cells of total memory CD8^+^ T cells for MS cohorts for total EBV (A), total CMV (B), or EBV stratified for latent and lytic peptides (C). (D) Percentage of EBV- or CMV-specific CD8^+^ T cells of total memory CD8^+^ T cells in a paired manner. (E) Differentiation stages of EBV- and CMV-specific CD8^+^ T cells for three cohorts. (F) Differentiation stages of EBV-specific CD8^+^ T cells restricted to latent or lytic peptides for three cohorts. Kruskal-Wallis test with Dunn posthoc (A, B, C, E, and F) or Wilcoxon test (D) was used for statistical testing. Every dot represents one individual. Bars represent medians (A–C) and lines show paired-wise analysis from the same donor (D). ∗*p* < 0.05, ∗∗*p* < 0.01, ∗∗∗*p* < 0.001, ns = not significant. See also Figures S2 and S3.

To study whether HLA-B7 and HLA-A2 differentially impact the virus-specific T cells in the context of MS, we investigated the frequencies and differentiation state of EBV- and CMV-specific CD8^+^ T cells from pwMS. No significant differences in frequencies of EBV- and CMV-specific memory CD8^+^ T cells were found between the healthy donors and pwMS, for both untreated MS (UT-MS) or natalizumab-treated MS (NTZ-MS) (Figures 2A–2C). The frequencies of all separate epitope-specific CD8^+^ T cells are shown in Figure S3. When comparing the percentages of EBV- and CMV-specific CD8^+^ T cells in HLA-A2^+^B7^+^ positive donors in a paired manner, HLA-B7-restricted cells were more abundant than HLA-A2-restricted cells (Figure 2D). This was significant for the CMV-specific cells and a similar trend was seen for EBV latent epitope-specific cells. Next, we compared the differentiation state of EBV- and CMV-specific CD8^+^ T cells between HD and pwMS. For UT-MS, a 1.4-fold decrease in the effector CD8^+^ T cell compartment (EM + TEMRA) and a 2.1-fold increase in the CM compartment were detected for EBV, which was restored in the NTZ-MS group. This was significant for HLA-A2 and nearly significant for HLA-B7 (Figure 2E). No differences were found for CMV-specific CD8^+^ T cells in pwMS compared to HD. When stratified for lytic and latent epitopes, this increase in CM and decrease in the effector compartment was especially seen for EBV latent epitopes. A significant increase of CM T cells was accompanied by a significant decreased of the EM subset for the A2-restricted EBV-specific CD8^+^ T cells and the B7-restricted CD8^+^ T cells followed the same trend (Figure 2F). This could indicate migration to the CNS of the EM subset.

### CD20^dim^ and CXCR3 expression characterizes EBV-specific CD8^+^ T cells

**Figure 3:**
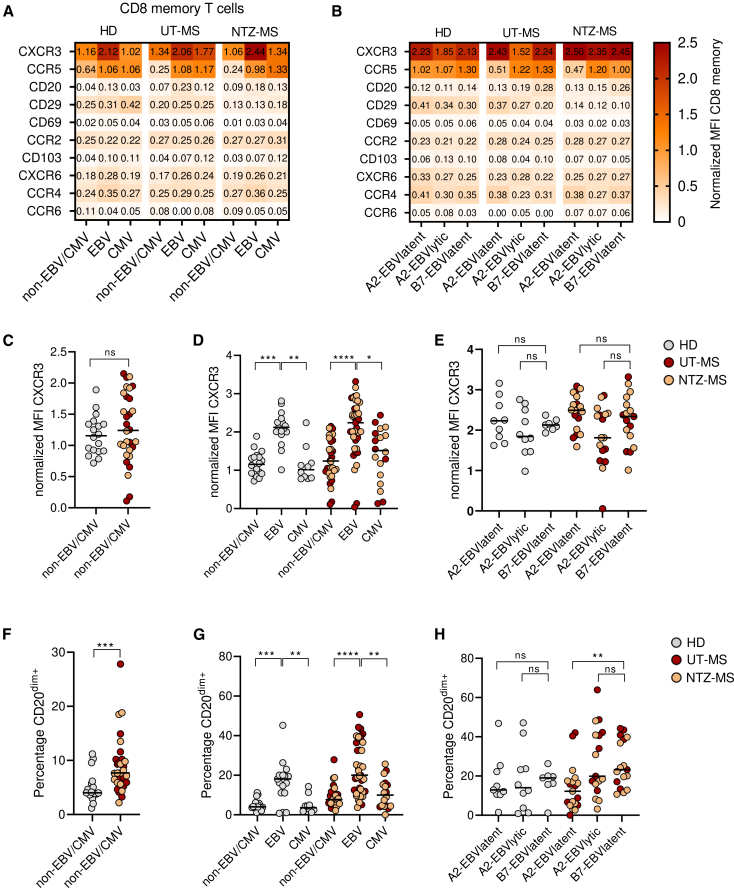
Tissue-homing and -residency-associated markers on EBV- and CMV-specific CD8^+^ T cells in HD and MS Heatmap of median protein expression in normalized MFI for non-EBV/CMV CD8^+^ memory T cells, EBV- and CMV-specific CD8^+^ memory T cells (A) or for EBV-specific CD8^+^ memory T cells restricted to HLA-A2 or HLA-B7 with latent or lytic peptides (B). CXCR3 expression for non-EBV/CMV CD8^+^ memory T cells (C), EBV- and CMV-specific CD8^+^ memory T cells (D) or for EBV-specific CD8^+^ memory T cells restricted to latent or lytic peptides (E). CD20^dim^ expression for non-EBV/CMV CD8^+^ memory T cells (F), EBV- and CMV-specific CD8^+^ memory T cells (G), or for EBV-specific CD8^+^ memory T cells restricted to latent or lytic peptides (H). Mann-Whitney test (C and F) or Kruskal-Wallis test with Dunn posthoc (D, E, G, and H) was used for statistical testing. Every dot represents one individual. Lines represent medians (C–H). ∗*p* < 0.05, ∗∗*p* < 0.01, ∗∗∗*p* < 0.001, ∗∗∗∗*p* < 0.0001, ns = not significant.. See also Figures S2, S4, and S5.

To further elaborate on the CNS-homing potential of EBV-specific memory CD8^+^ T cells, we evaluated tissue homing and residency associated markers (gating strategy is shown in Figure S2B). As we found a decrease of the EM subset in the UT-MS group, which could indicate migration to the brain, we investigated if the expression level of tissue homing and residency associated markers was higher on total CD8^+^ T_EM_ cells compared to the CM and TEMRA subsets. Indeed, CCR5, CD69, CCR2, CD103, and CXCR6 expression was higher in the EM subset compared to the CM or TEMRA subset in the UT-MS group. This was only found for CCR5 and CCR2 in HD and NTZ-MS group (Figure S4). This shows that the EM CD8^+^ T cells in the UT-MS group have the greatest potential to migrate toward to CNS. Next, we compared these markers between the groups on EBV- and CMV-specific CD8^+^ T cells. Except for the expected lower detection of CD29 on the CD8^+^ T cells from the NTZ-MS group due to the treatment, no differences in expression level of tissue-homing- and -residency-associated markers were found between the UT-MS and NTZ-MS groups for EBV- and CMV-specific CD8^+^ T cells, in median fluorescence intensity (MFI) (Figures 3A and 3B) and percentages (Figures S5A and S5B). Therefore, these data were combined for further analysis as one group. Furthermore, HLA-B7-restricted EBV lytic epitope-specific CD8^+^ T cells were excluded from the analysis since very few cells could be detected as shown in Figures 1 and 2. Samples with less than 20 epitope-specific CD8^+^ T cells were excluded from analysis as well. The expression level of CXCR3 was similar on CD8^+^ memory cells between HD and pwMS. However, for both groups, CXCR3 was increased on EBV-specific CD8^+^ memory T cells compared to non-EBV/CMV CD8^+^ memory T cells and CMV-specific CD8^+^ memory T cells (Figures 3C and 3D), which can be linked to the high expression of this marker on T cells residing in the CNS^7^ and high concentrations of its ligand CXCL10 in CSF.^12^ The differences in CXCR3 expression between EBV- and CMV-specific cells could at least be explained by different expression between memory subsets (Figure S4). This high CXCR3 expression on EBV-specific CD8^+^ T cells was not affected by restriction to HLA-B7 or HLA-A2 (Figure 3E). Furthermore, CD20^dim^ expression is enriched on circulating CD8^+^ T cells in pwMS, which was confirmed in the cohort of the current study (Figure 3F), and is even further upregulated on T cells residing in CNS tissues.^13^^,^^14^ Interestingly, CD20^dim^ expression was increased on EBV-specific CD8^+^ T cells compared to non-EBV/CMV CD8^+^ memory T cells and CMV-specific CD8^+^ T cells, which was found for both HD and pwMS (Figure 3G). When looking into epitope-specificity and HLA restriction, CD20 expression was highest on HLA-B7- compared to HLA-A2-restricted EBV-specific CD8^+^ T cells in pwMS (Figure 3H). For all other tissue-homing or residency associated markers, no significant differences were found between the groups.

### EBV latent epitope-specific CD8^+^ T cells have a more exhausted phenotype when restricted to HLA-B7 compared to HLA-A2

**Figure 4:**
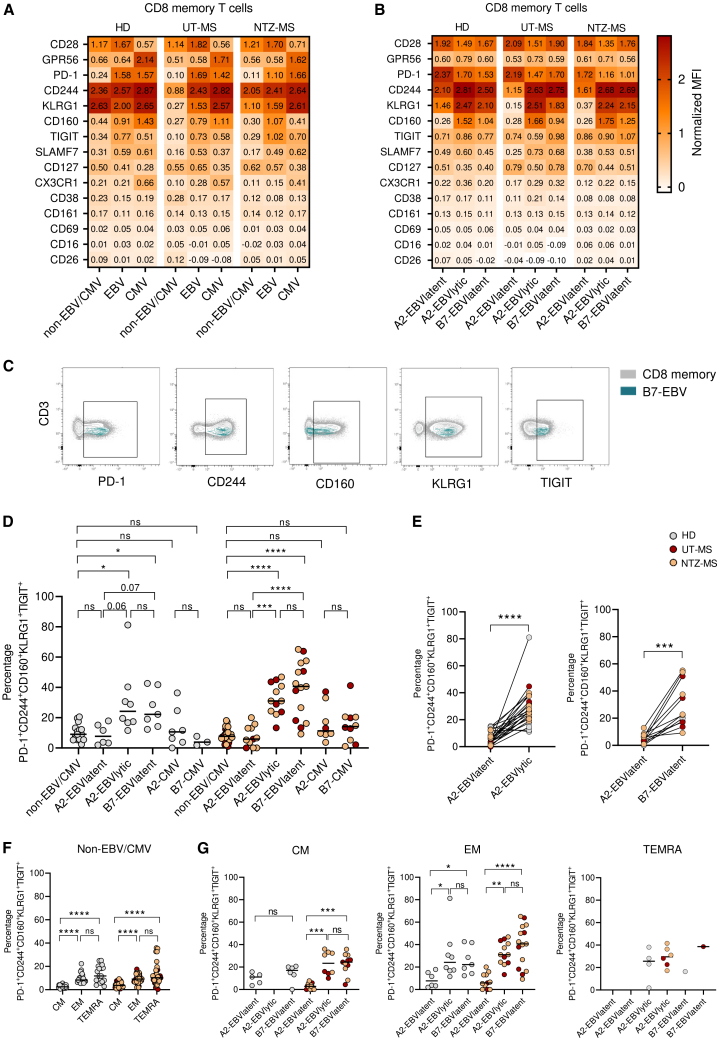
Activation-associated markers on EBV- and CMV-specific CD8^+^ T cells in HD and MS Heatmap of median protein expression in normalized MFI for non-EBV/CMV CD8^+^ memory T cells, EBV- and CMV-specific CD8^+^ memory T cells (A) or for EBV-specific CD8^+^ memory T cells restricted to HLA-A2 or HLA-B7 with latent or lytic peptides (B). (C) Representative example of gating of co-inhibitory receptors. (D) Exhaustion phenotype on EBV- and CMV-specific CD8^+^ memory T cells restricted to HLA-A2 or HLA-B7 with latent or lytic peptides. (E) Exhaustion phenotype on EBV-specific CD8^+^ memory T cells restricted to HLA-A2 or HLA-B7 with latent or lytic peptides in a paired manner. (F) Exhaustion phenotype on non-EBV/CMV CD8^+^ memory T cells. (G) Exhaustion phenotype on EBV-specific CD8^+^ memory T cells restricted to HLA-A2 or HLA-B7 with latent or lytic peptides stratified by differentiation state. Kruskal Wallis test with Dunn posthoc (D, F, and G) or Wilcoxon test (E) was used for statistical testing. Every dot represents one individual. Lines represent medians (D, F, G) and lines show paired-wise analysis from the same donor (E). ∗*p* < 0.05, ∗∗*p* < 0.01, ∗∗∗*p* < 0.001, ∗∗∗∗*p* < 0.0001, ns = not significant.. See also Figures S2 and S5–S9.

To further characterize the effector phenotype of EBV-specific CD8^+^ T cells, we compared surface levels of activation markers, co-stimulatory receptors, co-inhibitory receptors and cytotoxicity-related markers between the HLA and epitope-dependent subgroups (gating strategy shown in Figure S2C). No differences in expression were found between the UT-MS and NTZ-MS groups in MFI (Figures 4A and 4B) and percentages (Figures S5C and S5D), so these data were again combined in further analysis. EBV-specific CD8^+^ T cells were distinguished from CMV-specific CD8^+^ T cells by a higher expression of CD28, TIGIT, and CD127 (IL-7R) and a lower expression of GPR56, CX3CR1, and KLRG1 (Figures 4A and S6A–S6F), which is in line with previous studies.^15^ CD28 expression is significantly lower expressed and GPR56 expression is significantly higher expressed on TEMRA cells (Figure S7). Since a higher percentage of CMV-specific CD8^+^ T cells have a TEMRA phenotype compared to EBV-specific CD8^+^ T cells, this could explain these differences. Co-inhibitory receptors CD244, PD-1, and CD160 were all higher expressed on both EBV- and CMV-specific compared to non-EBV/CMV CD8^+^ memory T cells. These differences were found for both the HD and MS group (Figures S6G–S6I). The differences in expression of co-stimulatory and co-inhibitory markers especially stood out when comparing the EBV-specific CD8^+^ T cells between the HLA and epitope-dependent subgroups (Figure 4B). Lower expression of co-stimulatory marker CD28 was found on CD8^+^ T cells specific for EBV lytic epitopes compared to latent epitopes for HLA-A2 (Figure S8). Moreover, when comparing EBV-specific CD8^+^ T cells between different HLA types, higher expression of co-inhibitory receptors CD244, KLRG1 and CD160 was found for HLA-B7- versus HLA-A2-restricted CD8^+^ T cells specific for EBV latent epitopes (Figure S8). HLA-B7-restricted CD8^+^ T cells specific for EBV latent epitopes showed a similar phenotype as the HLA-A2-restricted CD8^+^ T cells specific for EBV lytic epitopes, except for a higher TIGIT expression (Figure S8). As co-expression of co-inhibitory receptors is one of the hallmarks of T cell exhaustion, we subsequently evaluated the co-expression of PD-1, CD244, CD160, KLRG1, and TIGIT. A representative example of the gating of each co-inhibitory receptor is shown in Figures 4C and S2C. A higher percentage of HLA-B7-restricted EBV-specific CD8^+^ T memory cells and HLA-A2-restricted EBV lytic epitope restricted CD8^+^ T memory cells had an exhausted phenotype compared to the non-EBV/CMV specific CD8^+^ T memory cells in both HD and pwMS (Figure 4D). In contrast, HLA-A2-restricted EBV latent epitope restricted CD8^+^ T memory cells and CMV-specific CD8^+^ T memory cells did not show this phenotype. Co-expression of these co-inhibitory receptors was most distinctive in pwMS and tended to be highest in HLA-B7-restricted CD8^+^ T cells (Figure 4D). These differences in co-expression on HLA-restricted and epitope-specific CD8^+^ T cells were also observed in a paired manner within the same individuals (Figure 4E). As expected, the expression of co-inhibitory receptors (PD1, CD244, KLRG1, CD160, and TIGIT) is higher on T_EM_ and/or T_EMRA_ cells compared to T_CM_ (Figure S7), as well as the co-expression of these markers (Figure 4F). Since HLA-A2-restricted T cells that recognize EBV latent epitopes contain more T_CM_ cells compared to the cells recognizing EBV lytic epitopes, this could influence our results. However, this is not the case as these differences are still observed when the CD8^+^ T cells were stratified for the CM, EM, and TEMRA phenotype (Figure 4G). Together, these results show that HLA-B7-restricted EBV-specific T cells have more exhausted phenotype and might be less able to control EBV even during the latent phase of the infection. To investigate if HLA-B7-restricted EBV-specific CD8^+^ T cells are also functionally more exhausted than HLA-A2-restricted EBV-specific CD8^+^ T cells, we measured proliferation and cytokine secretion after EBV peptide stimulation. We observed an increased percentage of CD8^+^ T cells in P5 gate, where the cells proliferated the most, after EBV peptide stimulation compared to actin peptide stimulation. However, we did not observe differences in proliferation between stimulation with the A2 EBV peptide pool or B7 EBV peptide pool (Figures S9A and S9B). Similarly, an increased percentage memory CD8^+^ T cells secreted IL-2, TNFα and IFNγ after EBV peptide stimulation. However, no differences were observed between stimulation with the A2 EBV latent peptide pool, A2 EBV lytic peptide pool or B7 EBV latent peptide pool (Figure S9C).

### MS CNS-compartments contain HLA-B7-restricted EBV-specific CD8^+^ T cells

**Figure 5:**
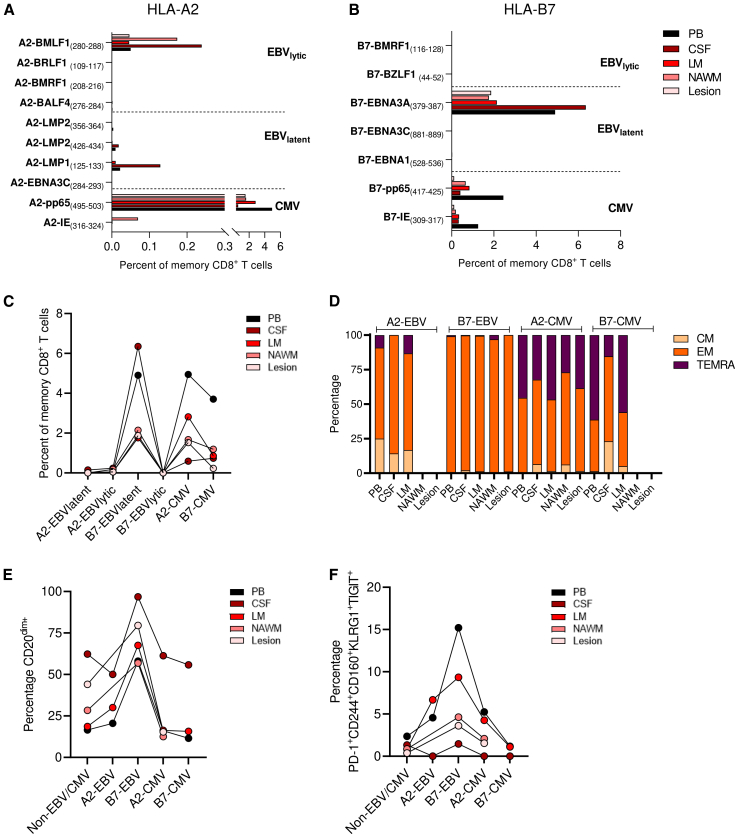
EBV- and CMV-specific CD8^+^ T cells in CNS compartments Percentage of EBV- and CMV-specific CD8^+^ T cells of total memory CD8^+^ T cells in blood and CNS compartments per immunodominant epitope restricted to HLA-A2 (A) or HLA-B7 (B). (C) Percentages of EBV-specific CD8^+^ T cells of total memory CD8^+^ T cells restricted to HLA-A2 or HLA-B7 with latent or lytic peptides in blood and CNS compartments. (D) Differentiation states of EBV- and CMV-specific CD8^+^ T cells in blood and CNS compartments. (E) Expression of CD20^dim^ of EBV- and CMV-specific CD8^+^ T cells in blood and CNS compartments. (F) Exhaustion phenotype of EBV- and CMV-specific CD8^+^ T cells in blood and CNS compartments. Peripheral blood (PB), cerebrospinal fluid (CSF), leptomeninges (LM), and normal appearing white matter (NAWM). Lines show paired-wise analysis from the same donor (C, E, F). See also Figures S10 and S11.

To explore how HLA restriction determines the presence of EBV-specific CD8^+^ T cells in the MS CNS, we isolated lymphocytes from postmortem CSF, leptomeninges (LM), normal appearing white matter (NAWM) and affected white matter (lesion) of an MS donor carrying both HLA-B7 and HLA-A2. Representative gating of EBV- and CMV-specific CD8^+^ T cells restricted to HLA-A2 or HLA-B7 is shown in Figure S10. Both EBV- and CMV-specific CD8^+^ T cells could be detected in the blood and the majority of CNS compartments, but their distribution was dependent on the HLA restriction (Figures 5A and 5B). Notably, within the CNS compartments, HLA-B7-restricted EBV-specific CD8^+^ T cells were most abundantly found, which was vice versa for CMV-specific CD8^*+*^ T cells (Figure 5C). In line with our findings in pre-mortem blood and in contrast to CMV-specific counterparts, EBV-specific CD8^+^ T cells displayed predominantly an EM phenotype. We did not find any differences in memory state of EBV- or CMV-specific CD8^+^ T cells between the blood and different CNS compartments (Figure 5D). As expected, tissue-homing and residency associated markers were upregulated on total CD8^+^ memory T cells in all CNS compartments as compared to blood (Figure S11A). Interestingly, HLA-B7-restricted EBV-specific CD8^+^ T cells expressed the highest level of these markers, especially in the CSF. Within all compartments, tissue residency-associated markers CD20 and CXCR6 were selectively increased on HLA-B7-restricted EBV-specific CD8^+^ T cells (Figures 5E and S11A). In addition, the co-expression profile of co-inhibitory markers as observed for HLA-B7-restricted EBV-specific CD8^+^ T cells was seen in all compartments, but less pronounced within the CNS than the blood (Figure 5F). Of the CNS compartments, this co-expression was the highest in the LM, in which most B cells are found postmortem in MS.^16^ In conclusion, in this MS case, predominantly HLA-B7-restricted EBV-specific EM CD8^+^ T cells have recruited to the CNS, of which a minority shows an exhausted phenotype.

### HLA-B7 and HLA-A2 distribution influences anti-EBNA1 IgG serum levels

**Figure 6:**
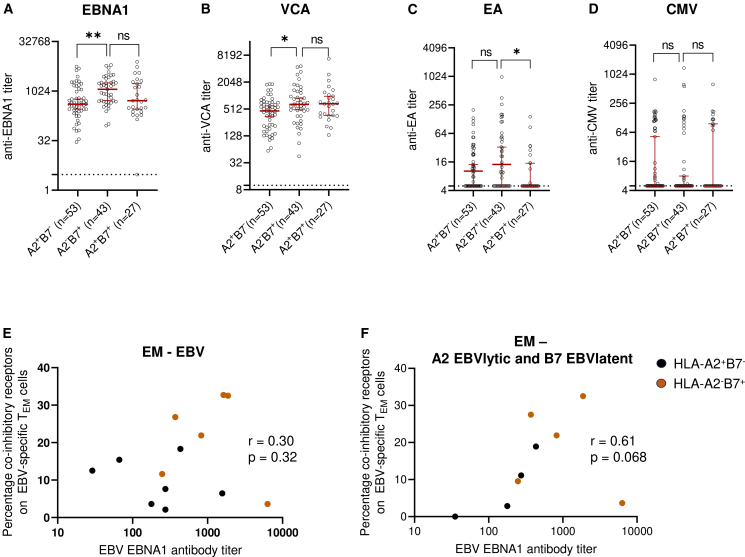
Correlation EBV antibody titers and exhaustion phenotype Antibody titers of HLA-A2^+^B7^-^, HLA-A2^-^B7^+^ or of HLA-A2^+^B7^+^ pwMS from the PROUD and MS-NTZ cohort. (A) EBNA1, (B) viral capsid antigen (VCA), (C) early antigen (EA), and (D) CMV. Kruskal-Wallis test was used for statistical testing. (E) Correlation of the percentage exhaustion phenotype on EBV-specific T_EM_ cells and EBNA1 antibody titer. UT-MS and NTZ-MS were included. (F) As in (E), but only A2-EBV lytic and B7-EBV latent specific cells are shown. Spearman correlation was used for statistical testing (E and F). Kruskal-Wallis test with Dunn posthoc was used for statistical testing (A–D) . Every dot represents one individual. Error bars show 95% confidence intervals (A–D). ∗*p* < 0.05, ∗∗*p* < 0.01, ns = not significant. See also Figure S12.

To connect HLA-B7 and/or HLA-A2 carriage with differential control of EBV infection, we measured serum antibody titers against EBV and CMV in all genotyped participants of the PROUD cohort with an RRMS diagnosis (*n* = 95) and all genotyped participants of the MS-NTZ cohort (*n* = 28). Antibody titers against EBV nuclear antigen 1 (EBNA1) and viral capsid antigen (VCA) were significantly higher in HLA-B7^+^HLA-A2^−^compared to HLA-B7^−^HLA-A2^+^ pwMS (Figures 6A and 6B) and antibodies against early antigen (EA) were significantly higher in HLA-B7^+^HLA-A2^-^ compared to HLA-B7^+^HLA-A2^+^ pwMS (Figure 6C). In contrast, no differences between the HLA types were found for anti-CMV antibodies (Figure 6D). Since MS risk allele HLA-DRB1∗15:01 is in linkage disequilibrium with HLA-B7 and could be responsible for these results, we also stratified these data accordingly. The co-occurrence of HLA-DRB1∗15:01 and HLA-A2 or HLA-B7 of selected samples for antibody titers are shown in Table S2. Indeed, if HLA-B7 was replaced by HLA-DRB1∗15:01, the same results were observed (Figures S12A–S12D). However, when the cohort was only stratified by HLA- DRB1∗15:01, only anti-VCA antibody levels were significantly higher in the HLA-DRB1∗15:01^+^ group (Figures S12E–S12G). Together, this highlights the protective effect of HLA-A2, lowering antibody levels for both anti-EBNA1 and anti-EA. Since high EBNA1 antibody levels are a known risk factor and showed the largest difference between HLA-A2 and HLA-B7, we assessed whether anti-EBNA1 antibody levels correspond to the effector phenotype of EBV-specific EM CD8^+^ T cells. We were able to measure the antibody level for 13 pwMS which were included in the spectral flow cytometry analysis and found a non-significant weak correlation between the exhaustion phenotype and EBNA1 antibody titer (Figure 6E). However, this correlation became stronger when selecting for HLA-A2-restricted EBV lytic epitope and HLA-B7-restricted EBV latent epitope-specific EM CD8^+^ T cells (Figure 6F), for which the exhaustion phenotype was most prominent (Figure 6D). This number of data points did just not meet significance, but the correlation nonetheless supports a model in which HLA-B7-restricted CD8^+^ T cells are more exhausted, and therefore, less capable of controlling an EBV infection.

## Discussion

The susceptibility to develop MS is multifactorial and includes many genetic and environmental risk factors. In this study, we focused on two prominent risk factors: EBV infection and HLA. We investigated the connection between these major risk factors by investigating EBV-specific CD8^+^ T cells restricted to HLA-B7 or HLA-A2. We show that EBV-specific CD8^+^ T cells restricted to HLA-B7 have a distinct effector and tissue-homing phenotype. This phenotype could not be explained by the linkage disequilibrium of HLA-B7 with HLA-DRB1∗15:01, since only very few samples in our study did not carry the HLA-DRB1∗15:01 allele. As exhausted T cells are less capable of controlling an EBV infection and this might lead to an aberrant immune response, this could explain why HLA-B7 carriers have a higher risk of developing MS compared to HLA-A2 carriers.

Our data show that, for both HD and pwMS, EBV-specific CD8^+^ T cell restricted to HLA-B7 recognized almost solely the latent epitope EBNA3A (379–387). This could lead to repeated antigen exposure for T cells restricted to HLA-B7, leading to the exhausted phenotype. Repeated antigen exposure and a pro-inflammatory environment could also potentially explain the skewing of HLA-B7-restricted CD8^+^ T cells recognizing EBNA3A (379–387) toward the EM phenotype. Although the most commonly used HLA-B7-restricted EBV epitopes were included, it remains possible that EBV-specific CD8^+^ T cells which recognize other epitopes are missed. EBV-specific CD8^+^ T cell restricted to HLA-A2 recognized a broader repertoire of latent and also lytic epitopes. This is in line with previous findings in HD where it has been shown that frequencies of A2-BMLF1-specific CD8^+^ T cells remain high in the latent phase of an EBV infection, while the frequencies of CD8^+^ T cells specific for other EBV-derived lytic epitopes decline.^17^ In the current study, EBV-specific CD8^+^ T cell frequencies were not different between HLA-A2 and HLA-B7 in HD and pwMS. This is variably shown in previous studies, where no differences, higher or lower frequencies were reported.^10^^,^^18^^,^^19^

In contrast to the similar frequencies, we did find phenotypic differences between CD8^+^ T cells recognizing EBV-derived lytic or latent epitopes and differences between EBV-specific CD8^+^ T cells recognizing epitopes presented by HLA-A2 or HLA-B7. In a previous single cell RNA-seq study, a higher expression of CD244 was found in HLA-A2 restricted CD8^+^ T cells specific for EBV-derived lytic versus latent epitope in HD,^9^ which is in line with our extended data on protein level. Additionally, we report that EBV-specific CD8^+^ T cells restricted to HLA-B7 (EBNA3A) likewise express more co-inhibitory receptors than the HLA-A2-restricted CD8^+^ T cells recognizing EBV latency epitopes. This difference was even more pronounced in pwMS than HD. The percentage of cells with this exhaustion phenotype was comparable between HLA-B7-restricted CD8^+^ T cells specific for EBV latent and HLA-A2-restricted CD8^+^ T cells specific for EBV lytic epitope. This implies that CD8^+^ T cells restricted to HLA-B7 become exhausted during the latent phase of an EBV infection, while this only occurs during the lytic phase for CD8^+^ T cells restricted to HLA-A2, when the viral load is higher. This is relevant as the disease often develops years after primary infection.^1^ We did not find any differences in our functional assays. However, a functional comparison between epitopes binding different HLA molecules is difficult to make. Different peptides will have different binding affinities which can have a major influence on the functional T cell response. Since we did not activate the T cells with phenotypic analysis, this approach is less prone to this bias. However, our phenotype does fit with reduced cytokine expression in pwMS compared to HD for HLA-B7-restricted EBV-specific CD8^+^ T cells published by Jilek et al.^10^ We show that the exhaustion phenotype is different between HLA-A2 and HLA-B7 in HD, likely contributing to the reduced risk of MS in HLA-A2 carriers. However, a lower cytokine expression in pwMS compared to HD has also been found for the protective allele HLA-A2,^18^ suggesting that more factors than HLA play a role in the impaired EBV-specific CD8^+^ T cell response in pwMS. Furthermore, Agostini et al. reported that a high EBV viral load in blood of pwMS is highly associated with HLA-B7.^11^ Reduced clearance of EBV by exhausted CD8^+^ T cells against EBV in HLA-B7^+^ individuals could lead to a higher EBV load. A subsequent increased antigen exposure to EBV-specific B cells could induce a higher antibody titer, is one of the hallmarks of MS. In line with this hypothesis, we found higher anti-EBNA1 titers in HLA-B7^+^ pwMS.

Next to the exhaustion phenotype, we investigated if HLA also plays a role in the phenotype of EBV-specific CD8^+^ T cells found in CNS compartments. Several studies have shown an enrichment of EBV-specific CD8^+^ T cells in the CSF and presence of these cells in the brain, which was confirmed in our study.^9^^,^^20^^,^^21^^,^^22^^,^^23^ Although we did not find an accumulation of total EBV-specific CD8^+^ T cells in the blood after natalizumab therapy, our data showed that mainly EBV-specific T_EM_ cells were restored in the blood after natalizumab therapy, suggesting that these cells migrate toward the brain. Indeed, T_EM_ cells showed the most tissue-homing and residency markers and the vast majority of the CD8^+^ T cell in the brain showed the EM phenotype for HLA-B7-restricted T cells. However, this was not the case for HLA-A2-restricted cells. We detected mainly HLA-B7-restricted EBV-specific CD8^+^ T cells in the brain, while Serafini et al. showed similar frequencies of HLA-A2 and HLA-B7-restricted EBV-specific CD8^+^ T cells.^22^ This might be explained by sample size, sample variation or differences in used techniques. Furthermore, we showed that EBV-specific CD8^+^ T cells express brain residency-associated marker CD20^13^^,^^14^ and that this expression remained high on these cells in the different CNS compartments, together with the expression of CXCR6. This phenotype of T cells in the brain is previously described and suggests a higher cytotoxic potential.^13^ A high cytotoxic potential of EBV-specific CD8^+^ T cells in the brain was previously suggested,^22^ highly contrasting with the exhaustion profile found in circulating HLA-B7-restricted EBV-specific CD8^+^ T cells. However, the exhausted phenotype was far less prominent in the brain compartments compared to the blood. This might be explained by the association of EBV with susceptibility of developing MS, while the association with MS severity remains controversial.^1^^,^^24^ Genetic SNPs in loci associated with HLA and adaptive immune responses likewise robustly associate with MS risk, while the influence of these factors on MS severity is uncertain.^25^ Thus, the reduced EBV control in the circulation for HLA-B7^+^ individuals could potentially be more important for susceptibility to MS than for control of EBV in the CNS in relation to MS pathology. As for the linkage disequilibrium between HLA-B7 and HLA-DRB1∗1501 as MS risk profile, the current thought is that during a primary infection, EBV-infected B cells first escape from CD8^+^ T cell-mediated control and then can receive more CD4^+^ T cell help to contribute to the pathogenesis in individuals carrying these alleles. The presence of CD8^+^ EBV-specific T cells in the CNS can have multiple explanations. This can be antigen-driven as it has been recently shown that EBV infected B cells can migrate to the brain and attract T cells.^26^ Furthermore, it has been shown that CXCL10, a ligand of CXCR3, is increased in the CSF of pwMS,^12^ which could attract the EBV-specific CD8^+^ T cells. Within the CNS, these EBV-specific CD8^+^ T cells could recognize infiltrated EBV-infected B cells, create a pro-inflammatory environment and thereby contribute to pathogenesis.

Since EBV is instrumental to the development of MS, preventive strategies could be applied targeting the EBV infection prior to MS onset. An important option would be prophylactic EBV vaccines. Although clinical trials have been performed and are planned, the development of these vaccines prove to be challenging.^27^^,^^28^ Since persons living with HIV have a reduced risk on developing MS, treatment with highly active anti-retroviral therapy has been hypothesized to prevent MS-onset in high risk populations.^29^ However, because of the relative low incidence of MS, possible negative health-effects of these drugs and their high costs, this hypothesis needs further investigation. Therapeutic strategies targeting EBV could also be an option as a benefit of reducing EBV viral load in patients with MS cannot be excluded. Anti-CD20 therapies deplete circulating memory B cells and are highly effective in pwMS. As B cells are the major target cells of EBV in human body, anti-CD20 therapy also depletes EBV, which might be part of the beneficial result.^30^ Another strategy is to adoptively transfer EBV-specific CD8^+^ T cells. Indeed, the first clinical trial with adoptive transfer of *in vitro*-expanded autologous EBV-specific CD8^+^ T cells has been performed with promising results.^31^ However, this strategy will not overcome T cell exhaustion and could maybe be replaced by TCR gene transfer therapy in the future.

To conclude, our data support the hypothesis that HLA-B7-restricted EBV-specific CD8^+^ T cells are more exhausted, and therefore, less able to control an EBV infection.^18^ Furthermore, these cells are able to cross the blood-brain barrier in pwMS as these cells were found in the brain. This could be key for the susceptibility to develop MS.

### Limitations of the study

There are a few limitations in this study. First, the number of subjects was limited due to the strict selection criteria and availability of the samples. All HD and pwMS needed to be selected first on HLA-A2 and HLA-B7. Furthermore, since CD8^+^ EBV-specific T cells are rare and the dominant antigen differs per person, not every sample and specificity reached our cut-off of 20 cells to continue phenotypical analysis. Therefore, we were unable to investigate the phenotype of HLA-B7 restricted CD8^+^ T cells specific for EBV lytic epitopes. It would be interesting to investigate if the exhaustion phenotype is also prominent in these cells. For the CNS compartments, we were able to include one donor. This can be considered as an anecdotal case, of which larger numbers have to be collected in time to validate results. Secondly, as this is a cross-sectional study, we cannot draw conclusions about the CD8^+^ EBV-specific T cells in the course of the disease. Furthermore, the tissue-homing and residency associated markers found on the EBV-specific CD8^+^ T cells are phenotypic characteristics. We did not directly show migration, but the presence of the CD8^+^ EBV-specific T cells in the different brain compartments indicate that these are able to migrate toward the brain. Lastly, we used mainly flow cytometry to investigate our focused hypothesis. Multi-omics might give a broader insight in the differences between EBV-specific CD8^+^ T cells restricted to different MS-associated HLA.

## Resource availability

### Lead contact

Requests for further information and resources should be directed to and will be fulfilled by the lead contact, Marvin van Luijn (m.vanluijn@erasmusmc.nl).

### Materials availability

There are restrictions to the availability of the HLA class I tetramers because of the lack of an external centralized repository for its distribution and our need to maintain the stock. We are glad to share the HLA class I tetramers with reasonable compensation by requestor for its processing and shipping.

### Data and code availability

• Data will be made available by the lead contact upon request. For sharing individualized data or material of participants who provided consent for sharing, a data transfer agreement (DTA) or material transfer agreement (MTA) needs to be developed as appropriate.
• This study does not report original code.
• Any additional information required to reanalyze the data reported in this study are available from the lead contact upon request.

## Acknowledgments

10.13039/501100003000Stichting MS Research (21-1142, 23-490g), Nationaal MS Fonds (OZ2021-016, P2021-001), MoveS stichting klimmen tegen MS. This project has received funding from European Union’s Horizon Europe Research and Innovation Actions under grant no. 101137235 (BEHIND-MS) as well as the Swiss State Secretariat for Education, Research and Innovation (SERI). Views and options expressed are, however, those of the authors only and do not necessarily reflect those of the European Union or the granting authorities. Neither the 10.13039/501100000780European Union nor the granting authorities can be held responsible for them.

The graphical abstract was created in BioRender. https://BioRender.com/21mwqof.

We thank Vera Middendorp for her work on the proliferation data during her internship, Jard de Vries from the department of Internal Medicine of the Erasmus MC for the HLA imputation, Dr. Corine H. Geurts van Kessel for supervising serological testing at the department of Viroscience of the Erasmus MC for the serology, and Peter van Geel for assistance at the Flow cytometry shared facility of the Erasmus MC.

## Author contributions

Conceptualization, S.R., R.N., J.S., and M.M.v.L.; investigation, S.R., J.R., A.M.M., A.F.W.-W., H.d.W., M.-J.M., and J.v.L.; visualization, S.R., A.M.M., and J.R.; statistical analysis, S.R. and Y.v.H.; resources, C.E.A.C., Y.M.M., and B.H.A.W.; funding acquisition, C.E.A.C., J.S., and M.M.v.L.; supervision, J.S. and M.M.v.L.; writing – original draft, S.R.; writing – review and editing, S.R., J.R., A.M.M., A.F.W., M.-J.M., C.E.A.C., Y.v.H., Y.M.M., J.S., and M.M.v.L.

## Declaration of interests

J.S. received speaker and/or consulting fee of Biogen, Merck, Novartis, Roche, and Sanofi-Genzyme. J.S. is member of scientific advisory committee of stichting MS research. MvL received research support from EMD Serono, Merck, Novartis, GSK, and Idorsia Pharmaceutical Ltd. M.M.v.L. is member of scientific advisory committee of National MS Fonds. The remaining authors declare no competing interests.

## STAR★Methods

### Key resources table

**Table:** 

REAGENT or RESOURCE	SOURCE	IDENTIFIER
**Antibodies**		

HLA-A2-PE (Clone BB7.2)	BD Biosciences	Cat#558570; RRID: AB_647220
HLA-B7-APC (Clone BB7.1)	BioLegend	Cat#372405; RRID: AB_2650775
CD28-BUV496 (Clone CD28.2)	BD Biosciences	Cat#741168; RRID: AB_2870741
GPR56-BUV563 (Clone CG4)	BD Biosciences	Cat#752709; RRID: AB_2917690
CD69-BUV737 (Clone RN50)	BD Biosciences	Cat#612818; RRID: AB_2870142
CD20-BUV805 (Clone 2H7)	BD Biosciences	Cat#612905; RRID: AB_2870192
CD196/CCR6-BV421 (Clone G034E3)	BioLegend	Cat#353408; RRID: AB_2561356
CD161-Pacific Blue (Clone HP-3G10)	BioLegend	Cat#339926; RRID: AB_2563960
CD192/CCR2-BV480 (Clone 1D9)	BD Biosciences	Cat#747852; RRID: AB_2872314
CD19-eFluor506 (clone HIB19)	Thermo Fisher Scientific	Cat#69-0199-42; RRID: AB_2637384
CD8-cFluor V547 (Clone SK1)	Cytek	Cat#R7-20063
CD127-BV570 (Clone A019D5)	BioLegend	Cat#351308; RRID: AB_2832685
CD194/CCR4- BV605 (Clone L291H4)	BioLegend	Cat#359417; RRID: AB_2562482
CX3CR1-BV650 (Clone 2A9-1)	BioLegend	Cat#341626; RRID: AB_2716245
CD195/CCR5-BV711 (Clone 2D7)	BD Biosciences	Cat#563395; RRID: AB_2738179
CD29-BV750 (Clone MAR4)	BD Biosciences	Cat#747231; RRID: AB_2871953
CD197/CCR7-BV785 (Clone G043H7)	BioLegend	Cat#353230; RRID: AB_2563630
CD160-AF488 (Clone BY55)	BD Biosciences	Cat#562351; RRID: AB_11153688
CD3-AF532 (Clone UCHT1)	Thermo Fisher Scientific	Cat#58-0038-41; RRID: AB_11219069
CD16-NovaFluor 610-70S (Clone 3G8)	Thermo Fisher Scientific	Cat#H006T02B06; RRID:AB_2896552
CD319/SLAMF7-BB700 (Clone 235614)	BD Biosciences	Cat#749692; RRID: AB_2873946
CD56-PerCP-eFluor710 (Clone TULY56)	Thermo Fisher Scientific	Cat#46-0566-42; RRID: AB_2637487
CD103-RB780 (Clone BER-ACT8)	BD Biosciences	Cat#569331; RRID: AB_3684981
CD186/CXCR6-RY586 (Clone 13B 1E5)	BD Biosciences	Cat#753575; RRID: AB_3687262
CD183/CXCR3-PE-Dazzle594 (Clone G0125H7)	BioLegend	Cat#353736; RRID: AB_2564288
PD-1-PE-Cy5 (Clone EH12.2H7)	BioLegend	Cat#329971; RRID: AB_2910394
CD244/2B4-PE-Cy5.5 (Clone C1.7)	Thermo Fisher Scientific	Cat#35-5838-42; RRID: AB_2784679
KLRG1-PE-Fire810 (Clone SA231A2)	BioLegend	Cat#367733; RRID: AB_2894622
CD4-AF647 (Clone SK3)	BioLegend	Cat#344635; RRID: AB_2566031
CD45RA-SparkNIR685 (Clone HI100)	BioLegend	Cat#304167; RRID: AB_2860793
CD26-AF700 (Clone 222113)	R&D Systems	Cat#FAB1180N; RRID: AB_3646032
TIGIT-APC-Cy7 (Clone A15253G)	BioLegend	Cat#372733; RRID: AB_2876700
CD38-APC-Fire810 (Clone HIT2)	BioLegend	Cat#303550; RRID: AB_2860784
IL-2-BV421 (Clone MQ1-17H1)	BioLegend	Cat#500328; RRID: AB_10962947
TNFα-BV750 (Clone Mab11)	BioLegend	Cat#502959; RRID: AB_3097294
IFNγ-RB613 (Clone B27)	BD Biosciences	Cat#571095; RRID: AB_3686229
Human TruStain FcX	BioLegend	Cat#422302; RRID: AB_2818986

**Bacterial and virus strains**		

HLA-A∗02:01 and HLA-B∗07:02 plasmids	John Altman, NIH Tetramer Facility, Emory University^32^	https://tetramer.yerkes.emory.edu
*Escherichia coli* BL21 (DE3)	Sigma-Aldrich	Cat#69450

**Biological samples**		

Peripheral blood heathy donor	Department of Immunology, Erasmus MC, Rotterdam	N/A
Peripheral blood and serum pwMS	Department of Neurology, Erasmus MC, Rotterdam	N/A
CNS compartments person with MS	The Netherlands Brain Bank	N/A

**Chemicals, peptides, and recombinant proteins**		

BMLF1(280-288)	JPT peptide technologies	N/A
BRLF1(109-117)	JPT peptide technologies	N/A
BMRF1(208-216)	JPT peptide technologies	N/A
BALF4(276-284)	JPT peptide technologies	N/A
LMP2(356-364)	JPT peptide technologies	N/A
LMP2(426-434)	JPT peptide technologies	N/A
LMP1(125-133)	JPT peptide technologies	N/A
EBNA3C(284-293)	JPT peptide technologies	N/A
pp65(495-503)	JPT peptide technologies	N/A
IE(316-324)	JPT peptide technologies	N/A
BMRF1(116-128)	JPT peptide technologies	N/A
BZLF1(44-52)	JPT peptide technologies	N/A
EBNA3A(379-387)	JPT peptide technologies	N/A
EBNA3C(881-889)	JPT peptide technologies	N/A
EBNA1(528-536)	JPT peptide technologies	N/A
pp65(265-275)	JPT peptide technologies	N/A
pp65(417-425)	JPT peptide technologies	N/A
IE(309-317)	JPT peptide technologies	N/A
Streptavidin-BUV395	BD Biosciences	Cat#564176; RRID: AB_2869553
Streptavidin-BUV661	BD Biosciences	Cat#612979; RRID: AB_2870251
Streptavidin-PE	Thermo Fisher Scientific	Cat#S866
Streptavidin-PE-Cy7	BioLegend	Cat#405206
Streptavidin-APC	Thermo Fisher Scientific	Cat#SA1005
Collagenase type IV	Thermo Fisher Scientific	Cat#17104019
DNase I recombinant	Roche	Cat#04536282001

**Critical commercial assays**		

Fixable Viability Kit-Zombie NIR	BioLegend	Cat#423106
LIAISON® EBNA IgG	Diasorin	Cat#310520
LIAISON® EA IgG	Diasorin	Cat#310540
LIAISON® VCA IgG	Diasorin	Cat#310510
LIAISON® Cytomegalovirus (CMV) IgG	Diasorin	Cat#310745

**Software and algorithms**		

HLA-TAPAS algorithm	Luo et al.^33^	https://github.com/immunogenomics/HLA-TAPAS
OMIQ	Dotmatics	https://www.omiq.ai/
Cytonorm	Van Gassen et al.^34^	https://github.com/saeyslab/CytoNorm
Graphpad Prism 9.2.0	Dotmatics	https://www.graphpad.com/

### Experimental model and study participant details

#### Study design, cohorts and selection criteria

The samples used for this study were selected from 3 cohorts consisting of 58 healthy donors (HD), 419 participants of the PROUD study (UT-MS) and 56 persons with RRMS treated with natalizumab (NTZ-MS). The PROUD study is a prospective cohort that includes persons after their first demyelinating event. MS diagnosis was made according to the McDonald 2017 criteria.^35^ Selection criteria for this study were sample availability and positivity for HLA-A2 and/or HLA-B7, resulting in 15 HD, 15 untreated pwMS and 15 NTZ-treated pwMS for spectral flow cytometry. All selected pwMS were positive for EBV-specific antibodies reactive towards EA, VCA and/or EBNA1. For the selected participants from the NTZ-MS cohort, the time of blood sampling was between 6 and 18 months after the first round of treatment. The sex, age and presence of HLA-A2, HLA-B7 and accompanying HLA-DRB1∗15:01 of the participants used for flow cytometry are described in Table S1. Information about gender, ancestry, race and ethnicity of participants was not available. It was not possible to properly analyze the influence of age or sex on study results due to our strict selection criteria and numbers of samples with sufficient numbers of virus-specific T cells. Fresh port-mortem blood and CNS tissues were obtained from an HLA-A2^+^B7^+^ MS patient (77 years, male) as part of the donor program of the Netherlands Brain Bank in Amsterdam.

#### Ethics statement

The PROUD study, ErasMS biobank and collection of the HD material were approved by the ethics committee of Erasmus MC, University Medical Center Rotterdam (MEC-2021-0946, MEC-2022-0389, MEC-2021-0251, respectively). Informed consent forms were signed by all participants. The Netherlands Brain Bank donor program was approved by the ethics committee of the Vrije Universiteit Medical Center, Amsterdam (2009/148).

### Method details

#### Isolation of mononuclear cells from peripheral blood

Peripheral blood was collected in CPT tubes, the PBMCs were isolated according to manufacturer’s protocol and stored in liquid nitrogen. Serum was collected and stored at -80 freezer. Peripheral blood from the MS brain donor was obtained post-mortem via cardiac puncture and PBMCs were isolated using Ficoll-Plaque Plus (GE Heathcare) as previously described.^6^

#### Isolation of mononuclear cells from post-mortem CNS compartments

Cerebrospinal fluid (CSF), leptomeninges (LM), normal appearing white matter (NAWM), and lesion from a person with MS were derived from the Netherlands Brain Bank. Mononuclear cells were isolated from the different compartments as previously described,^6^^,^^36^^,^^37^ with the addition of erythrocyte lysis and a modification for the enzymatic incubation step. Per 2-3 gram LM, NAWM or lesion, 7.5mg Collagenase type IV (Thermo Fisher Scientific) together with 500U DNAse I rec (Roche) in DPBS+Ca/Mg+0.1% BSA were added and incubated at 37°C for 60 min before mechanical dissociation.

#### HLA genotyping

For the UT-MS and NTZ-MS cohort, genomic DNA was isolated from blood samples and used for whole genome sequencing (Illumina Infinium High-Throughput Screening iSelect custom-730K SNP beadchip array). Based on these data, 4-digit HLA typing was performed by HLA imputation using the HLA-TAPAS algorithm.^33^ For the HD cohort and MS brain donor, genotyping for HLA-A2 and HLA-B7 was performed by flow cytometry. PBMCs were stained with HLA-A2-PE and HLA-B7-APC for 15 min at RT in the dark, washed with FACS buffer (PBS+0.5%BSA+0.01% Sodium Azide), and measured on a BD LSRFortessa Flow Cytometer (BD Biosciences). The used antibodies are listed in the key resources table. For the HD positive for HLA-B7 using flow cytometry but without detection of EBV-specific CD8 T cells, genotyping was performed to verify HLA-B7 positivity. This resulted in the exclusion of 1 HD who was HLA-B27 positive and HLA-B7 negative.

#### Peptides

A selection of the most reported immunodominant peptides based on the immune epitope database (iedb.org)^38^ were ordered at JPT peptide technologies (>70% purity, N-terminus amine, C-terminus acid, counter-ion trifluoroacetate), dissolved in MilliQ to a concentration of 1mg/ml and stored at -20°C till further use. All peptides are listed in the key resources table and Table S3.

#### EBV- and CMV-specific tetramers

EBV- and CMV- specific cells were identified using in house prepared tetramers. The HLA-A∗02:01 and HLA-B∗07:02 plasmids were received from John Altman, NIH Tetramer Facility at Emory University. HLA monomers were produced using *Escherichia coli* BL21 (DE3) competent cells and proteins were purified using an ÄKTA pure chromatography system (Cytiva). Tetramers were produced as previously described,^32^ with the modification that monomers were prepared with photocleavable peptides.^39^^,^^40^ EBV- and CMV-derived peptides were exchanged as previously described.^41^ In short, the monomers and peptides were diluted in exchange buffer (20mM Tris + 100mM NaCl, pH=8.0), mixed and exposed for 1 hour to UV light. A separate tube with monomers was used for every peptide. Next, fluorochrome-coupled streptavidin diluted in exchange buffer was added in 10 steps with 15 min incubations on RT in between. Every peptide-monomer combination was separately labeled with 2 different fluorochromes. Tetramers were stored at 4°C.

#### Flow cytometry

The spectral flow cytometry panel was designed with a focus on activation-associated markers and brain-associated markers. Frozen PBMCS were thawed, counted and 5x10ˆ6 PBMCs were used per staining. First, the cells were stained with Zombie NIR Fixable Viability Kit (BioLegend) for 10min at RT. Cells were washed once with FACS buffer and resuspended in Brilliant Stain Buffer (BSB; BD Biosciences). Fc Receptor blocking solution (Human TruStain FcX, BioLegend), antibodies against CCR7 and CCR2 were added and incubated 5 min at RT, followed by an antibody against CCR6 for 2 min at RT, CXCR6 and CCR4 antibodies for 2 min at RT, an antibody against CCR5 for 2 min at RT and an incubation of 20 min at RT after adding an antibody against CXCR3. The cells were washed twice with FACS buffer and stained with the HLA-A2 or HLA-B7 tetramer mix. For the HLA-A2^+^B7^+^ samples, 2 separate protocols were performed with each 5x10ˆ6 cells, one using tetramers for HLA-A2 and one for HLA-B7. After 15 min incubation at RT, cells were washed twice with FACS buffer. Subsequently, the following antibodies were added 1 by 1 in BSB and incubated 20 min at RT: CD28, GPR56, CD69, CD20, CD127, SLAMF7, CX3CR1 and CD29. Cells were washed twice with FACS buffer and stained with an antibody mix diluted in FACS buffer: CD4, CD161, CD19, CD8, CD160, CD3, CD56, CD16, CD244, KLRG1, CD26, PD-1, CD45RA, CD38, TIGIT and CD103. After 20 min incubation at RT, cells were washed twice in FACS buffer and resuspended in PBS+0.1% BSA. The samples were measured on a Cytek Aurora 5 laser spectral cytometer. All antibodies are listed in the key resources table.

#### Proliferation assay

PBMCs were stained with 0.5μM CellTrace Violet (ThermoFisher) according to Manufacturer’s instructions. Afterwards, 5x10ˆ6 PBMCs per condition were cultured in RMPI+10% FCS+P/S in the presence of 1ng/ml IL-7 and IL-15 and 1μg/ml Actin peptide pool (PepMix human Actin, JPT), HLA-A2 EBV peptide pool (BMLF1(280-288), BMRF1(208-216), BRLF1(109-117), LMP2(356-364) and LMP2(426-434)) or HLA-B7 EBV peptide pool (BMRF1(116-128) and EBNA3A(379-387). After 6 days, the cells were stained with Zombie NIR Fixable Viability Kit (BioLegend) for 10min at RT. Cells were washed once with FACS buffer and stained with an antibody mix diluted in Brilliant Stain Buffer (BSB; BD Biosciences): CD3, CD4, CD8, CCR7 and CD45RA. After 20 min incubation at RT, cells were washed twice in FACS buffer and resuspended in PBS+0.1% BSA. The samples were measured on a Cytek Aurora 5 laser spectral cytometer. All antibodies and peptides are listed in the key resources table and in Table S3.

#### Cytokine assay

2x10ˆ6 PBMCs per condition were cultured in RMPI+10% FCS+P/S in the presence of 1μg/ml CD28 monoclonal antibody (CD28.2)(eBioscience) and 1μg/ml HLA-A2 EBVlatant peptide pool (LMP2(356-364), LMP2(426-434), EBNA3C(284-293) and LMP1(125-133)), HLA-A2 EBVlytic peptide pool (BMLF1(280-288), BMRF1(208-216), BRLF1(109-117) and BALF4(276-284)) or HLA-B7 EBVlatent peptide pool (EBNA3A(379-387, EBNA3C(881-889) and EBNA1(528-536)). 50ng/ml PMA (Merck) and 1μg/ml Ionomycin (Merck) was used as positive control. After 1 hour, 1μg/ml Brefaldin A (BioLegend) was added to the culture. After 5 additional hour of culture, the cells were stained with Zombie NIR Fixable Viability Kit (BioLegend) for 10min at RT. Cells were washed once with FACS buffer and stained with an antibody mix diluted in Brilliant Stain Buffer (BSB; BD Biosciences): CD3, CD4, CD8, CCR7 and CD45RA. After 20 min incubation at RT, cells were washed twice in FACS buffer and fixed and permeabilized using Manufacturer’s instructions (FoxP3 fixation/permeabilization concentrate and diluent, eBioscience). Subsequently, the cells were stained with IL-2, TNFα and IFNγ and measured on a Cytek Aurora 5 laser spectral cytometer. All antibodies and peptides are listed in the key resources table and in Table S3.

#### Serology

Serum samples from participants were collected at baseline for the PROUD study and 6 months to 7 years after treatment for the NTZ-MS cohort. Epstein-Barr virus (EBV) nuclear antigen EBNA-1 (NA), early antigen (EA) and viral capsid antigens (VCA) and Cytomegalovirus (CMV) IgG antibodies were measured using the LIAISON® IgG chemiluminescence immunoassays (CLIA) on the LIAISON® XL analyzer (Diasorin, Italy). The serological test and the analysis were conducted according to the manufacturer's instructions. The serum samples were 20x diluted to prevent results from exceeding the detection limit of the tests. To ensure reliability, quality control samples supplied by the manufacturer were included in each testing batch.

### Quantification and statistical analysis

#### Data analysis

Flow cytometry data were analyzed using OMIQ software form Dotmatics. EBV- and CMV specific T cells were gated manually and later pooled as EBV-latent, EBV-lytic or CMV-specific cells. Reference PBMCs from the same donor were taken along in every experiment to adjust for technical variation in marker expression. The expression level of the markers in the samples were normalized with CytoNorm using the reference PBMCs.^34^ Samples with fewer than 20 epitope-specific cells in a group were excluded from phenotypic analysis. For the brain compartments, CX3CR1 was used to exclude microglia (Figure S10A).

#### Statistical analysis

Statistical analysis was done in Graphpad Prism 9.2.0. The most suitable statistical tests were chosen based on the distribution and variance in the data and are described in the figure legend. A P value lower than 0.05 was considered significant. Every dot in the graphs represents an individual, *n* represents amount of individuals, the bar heights or lines represent the medians.
