# Supplementary material for: Multiple sclerosis-associated HLA demarcates EBV-specific CD8+ T cells with an exhausted and brain residency phenotype
Source: iScience. 2026 Apr 15;29(5):115744. doi: 10.1016/j.isci.2026.115744 (PMC13157025; doi:10.1016/j.isci.2026.115744)
Supplement: Document S1. Figures S1–S12 and Tables S1–S3 [file mmc1.pdf]

iScience, Volume *29*

Supplemental information

Multiple sclerosis-associated HLA demarcates EBV-specific $CD8^+$ T cells with an exhausted and brain residency phenotype

Sanne Reijm, Ana M. Marques, Jasper Rip, Cato E.A. Corsten, Annet F. Wierenga-Wolf, Harm de Wit, Marie-José Melief, Yifan van Hasselt, Jamie van Langelaar, Rinze Neuteboom, Beatrijs H.A. Wokke, Yvonne M. Mueller, Joost Smolders, and Marvin M. van Luijn

iScience, Volume ■■

Supplemental information

Multiple sclerosis-associated HLA demarcates EBV-specific $CD8^{+}$ T cells with an exhausted and brain residency phenotype

Sanne Reijm, Ana M. Marques, Jasper Rip, Cato E.A. Corsten, Annet F. Wierenga-Wolf, Harm de Wit, Marie-José Melief, Yifan van Hasselt, Jamie van Langelaar, Rinze Neuteboom, Beatrijs H.A. Wokke, Yvonne M. Mueller, Joost Smolders, and Marvin M. van Luijn

SUPPLEMENTAL INFORMATION

Table S1. Characteristics of selected samples for flow cytometry, related to STAR methods.

	HD (n=15)	UT-MS (n=15)	NTZ-MS (n=15)
**Sex**			
Female, n (%)	8 (53.3)	9 (60)	12 (80)
Male, n (%)	7 (46.7)	6 (40)	3 (20)
**Age**			
Median	43	30	38
Range	25-69	25-50	23-62
**HLA types**			
$A2^+B7^-$, n (%)	6 (40)	5 (33.3)	4 (26.7)
$A2^-B7^+$, n (%)	5 (33.3)	5 (33.3)	4 (26.7)
$A2^+B7^+$, n (%)	4 (26.7)	5 (33.3)	7 (46.7)
$A2^+$DRB1*15:01$^+$, n (% of $A2^+$)	Unknown	6 (60)	10 (91)*
$B7^+$DRB1*15:01$^+$, n (% of $B7^+$)	Unknown	9 (90)	11 (100)

* DNA quality was not sufficient to perform HLA-DR15:01 genotyping (n=1)

Table S2. Co-occurrence of HLA-DRB1*15:01 and HLA-A*02 or HLA-B*07 of selected samples for antibody titers, related to Figure 6 and S12.

	UT-MS	NTZ-MS
**HLA types**		
$B7^+$DRB1*15:01$^+$, n (% of $B7^+$)	45 (88.2)	19 (86.4)
$B7^-$DRB1*15:01$^+$, n (% of $B7^-$)	28 (25.2)	9 (34.6)
$A2^+$DRB1*15:01$^+$, n (% of $A2^+$)	29 (44.6)	14 (82.4)
$A2^-$DRB1*15:01$^+$, n (% of $A2^-$)	44 (44.4)	14 (45.2)

Table S3. Peptides, related to STAR methods.

Peptide	Sequence	HLA restriction	Virus	Lytic or latent protein
BMLF1(280-288)	GLCTLVAML	HLA-A*02:01	EBV	Lytic
BRLF1(109-117)	YVLDHLIVV	HLA-A*02:01	EBV	Lytic
BMRF1(208-216)	TLDYKPLSV	HLA-A*02:01	EBV	Lytic
BALF4(276-284)	FLDKGTYTL	HLA-A*02:01	EBV	Lytic
LMP2(356-364)	FLYALALLL	HLA-A*02:01	EBV	Latent
LMP2(426-434)	CLGGLLTMV	HLA-A*02:01	EBV	Latent
LMP1(125-133)	YLLEMLWRL	HLA-A*02:01	EBV	Latent
EBNA3C(284-293)	LLDFVRFMGV	HLA-A*02:01	EBV	Latent
pp65(495-503)	NLVPMVATV	HLA-A*02:01	CMV	Lytic
IE(316-324)	VLEETSVML	HLA-A*02:01	CMV	Lytic
BMRF1(116-128)	RPQGGSRPEFVKL	HLA-B*07:02	EBV	Lytic
BZLF1(44-52)	LPCVLWPVL	HLA-B*07:02	EBV	Lytic
EBNA3A(379-387)	RPPIFIRRL	HLA-B*07:02	EBV	Latent
EBNA3C(881-889)	QPRAPIRPI	HLA-B*07:02	EBV	Latent
EBNA1(528-536)	IPQCRLTPL	HLA-B*07:02	EBV	Latent
pp65(265-275)	RPHERNGFTVL	HLA-B*07:02	CMV	Lytic
pp65(417-425)	TPRVTGGGAM	HLA-B*07:02	CMV	Lytic
IE(309-317)	CRVLCCYVL	HLA-B*07:02	CMV	Lytic

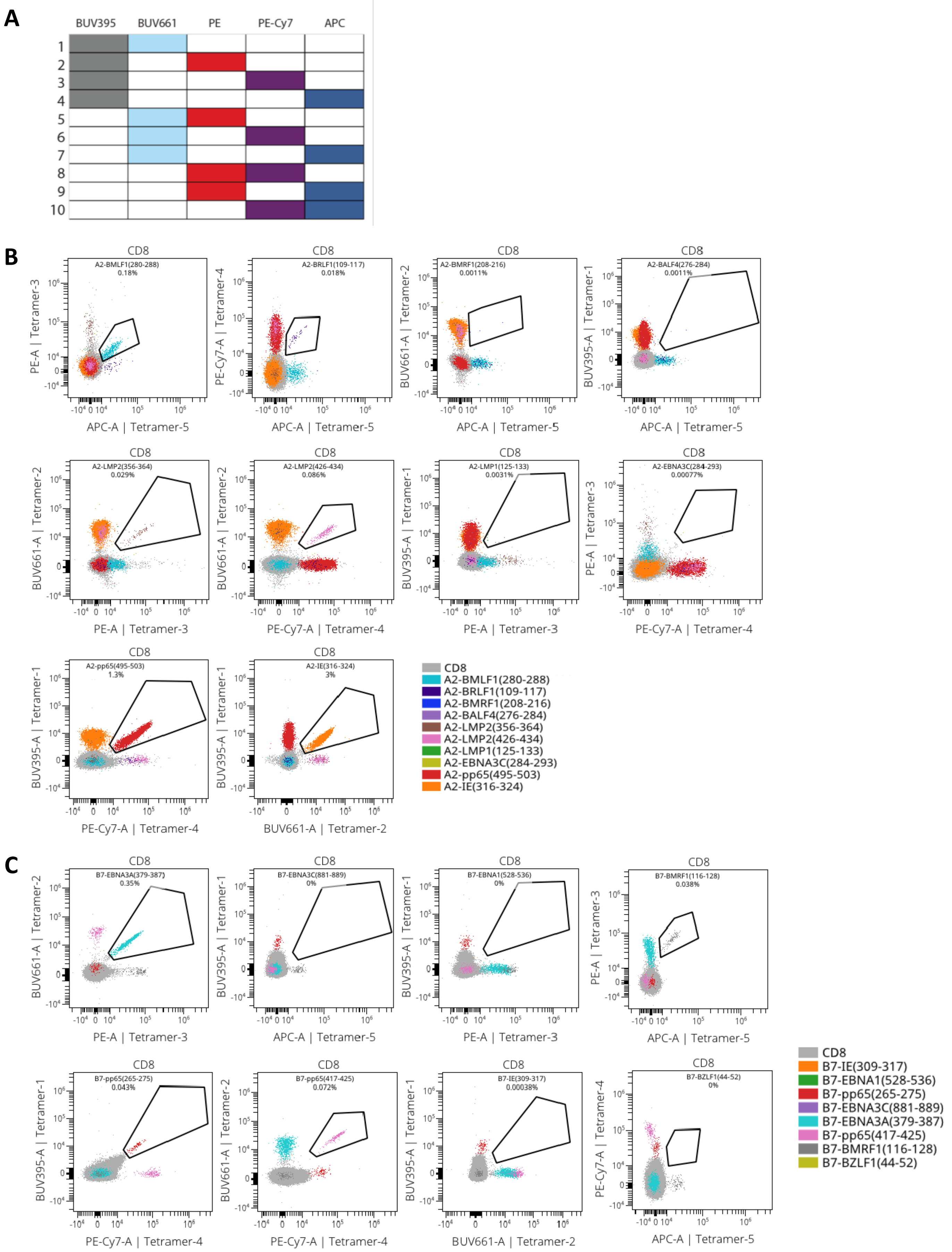

Figure S1. EBV- and CMV-specific CD8+ T cell gating, related to Figure 1. (A) Combinatorial staining methods for HLA class I tetramers The columns depict the 5 different fluorochromes used to conjugate tetramers. Each row depict an epitope-specific tetramer. **(B)** Representative example of a tetramers staining on a HD with HLA-A2 EBV- and CMV-tetramers. **(C)** Representative example of a tetramers staining on a HD with HLA-B7 EBV- and CMV-tetramers.

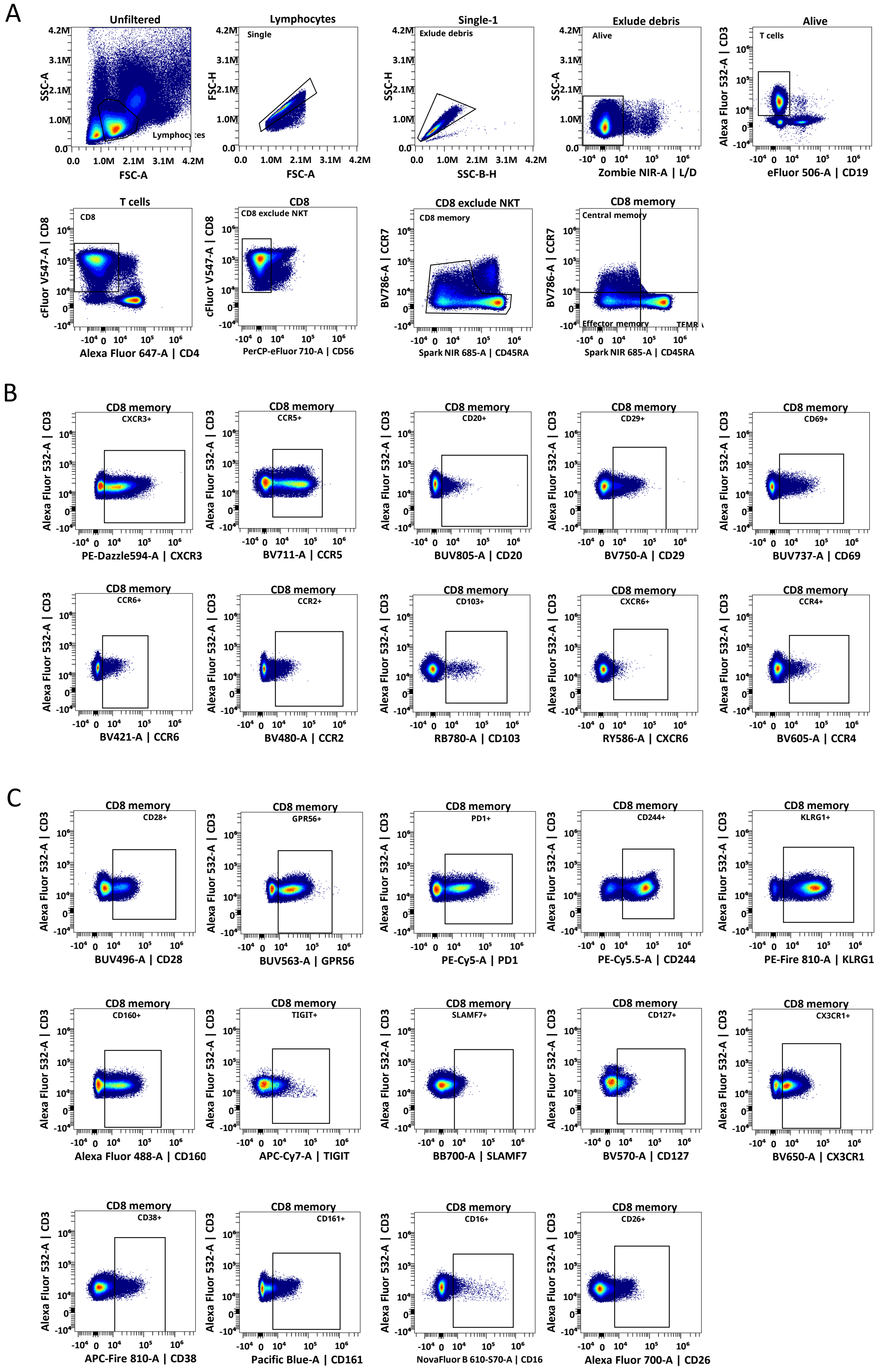

Figure S2. Gating strategy markers, related to Figure 1, 2, 3 and 4. (A) Representative gating strategy memory CD8+ T cells. **(B)** Representative gating tissue-homing/residency-associated markers or (**C**) activation-associated markers.

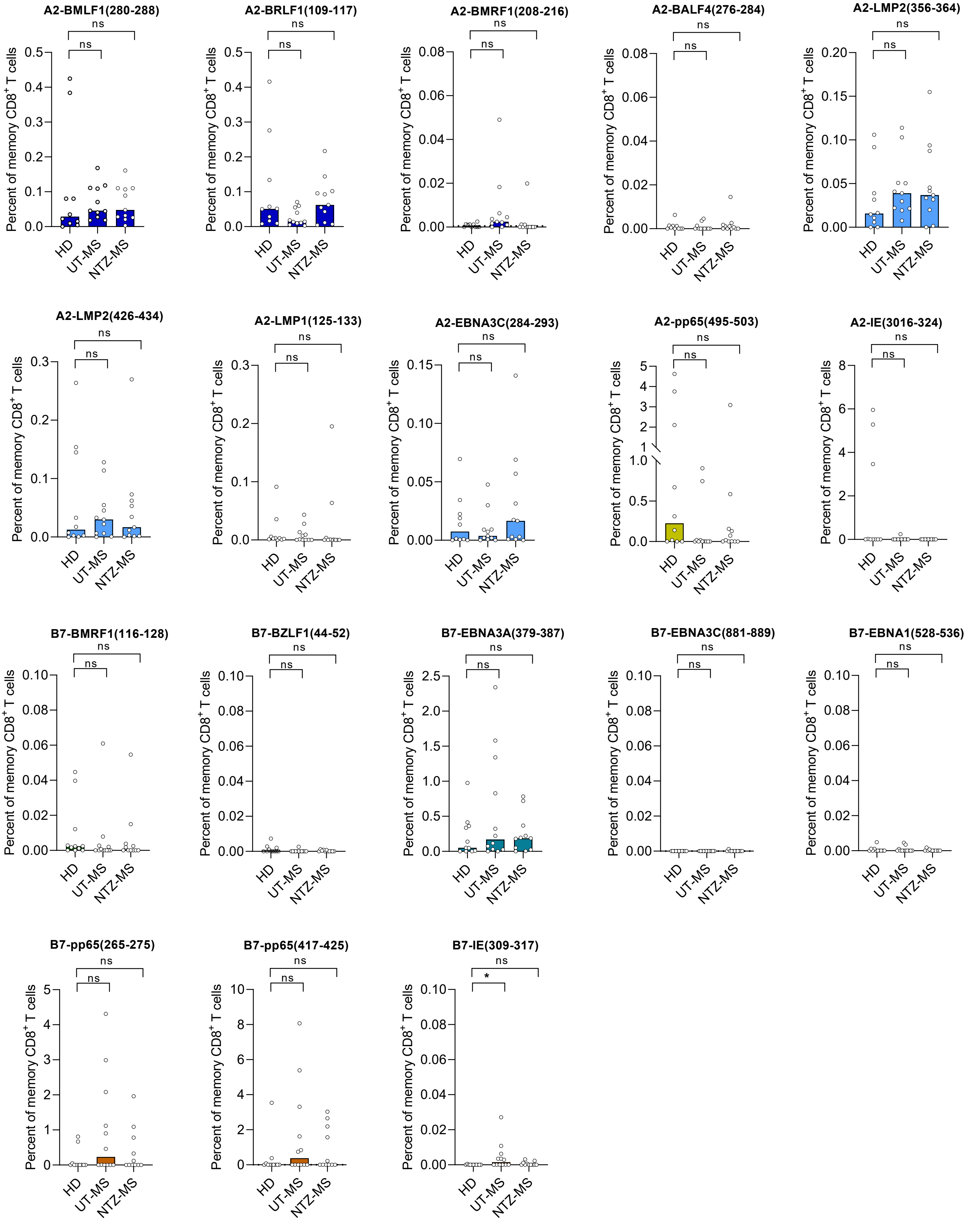

Figure S3. Frequencies of EBV- and CMV-specific CD8+ T cells in pwMS for every epitope, related to Figure 2. Every dot represents one individual. Bars represent median. Kruskal-Wallis test with Dunn posthoc was used for statistical testing. *p<0.05, ns = not significant.

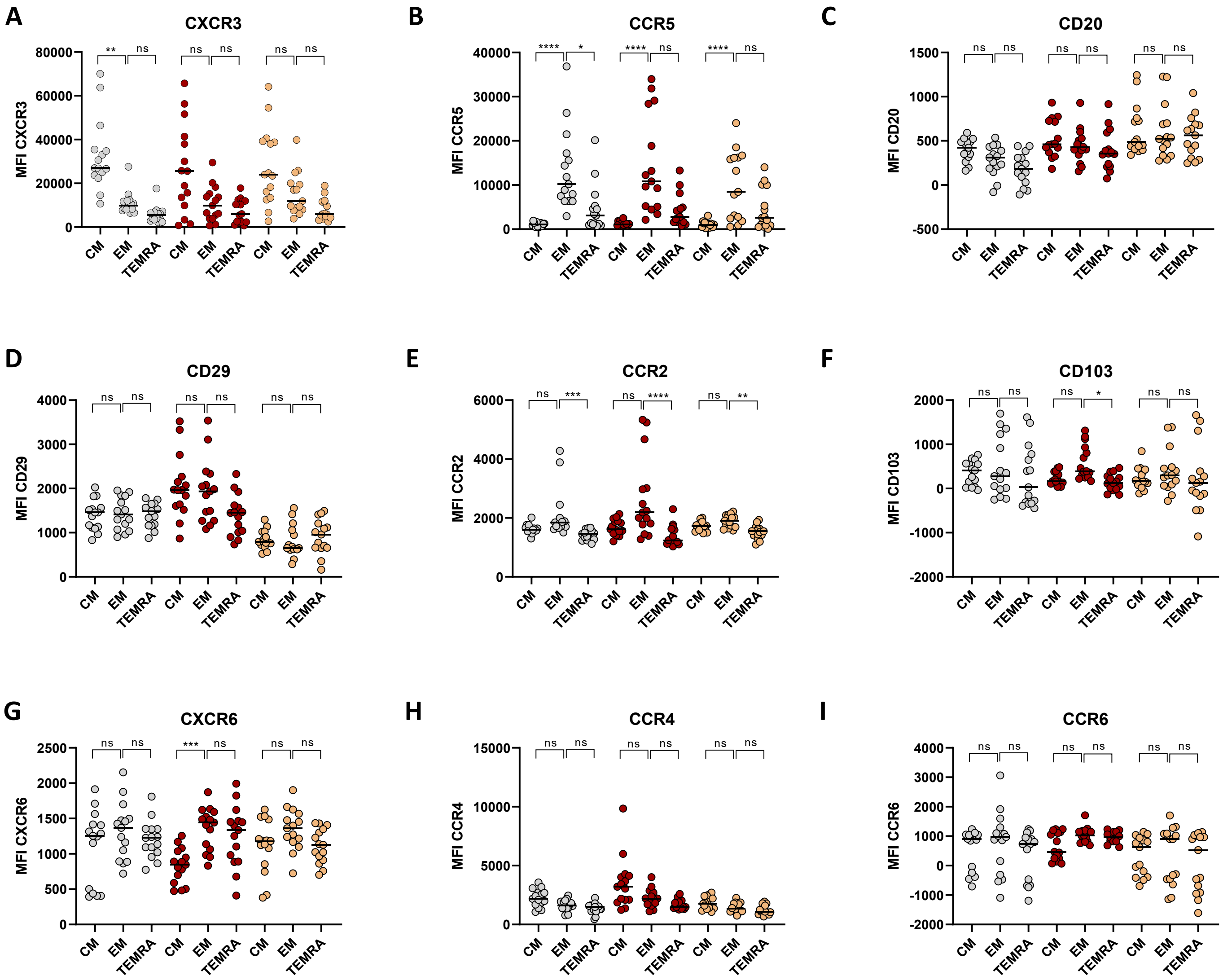

Figure S4. Differential marker expression between CD8⁺ T_{CM}, T_{EM} and TEMRA cells for tissue homing/residency-associated markers, related to Figure 3. Median Fluorescence (MFI) of CXCR3 **(A)**, CCR5 **(B)**, CD20 **(C)**, CD29 **(D)**, CCR2 **(E)**, CD103 **(F)**, CXCR6 **(G)**, CCR4 **(H)** and CCR6 **(I)**. Kruskal-Wallis test with Dunn posthoc was used for statistical testing. Every dot represents one individual. Grey dots represent HD, red dots UT-MS and orange dots NTZ-MS. Lines represent medians. *p<0.05, **p<0.01, ***p<0.001, ****p<0.0001, ns = not significant.

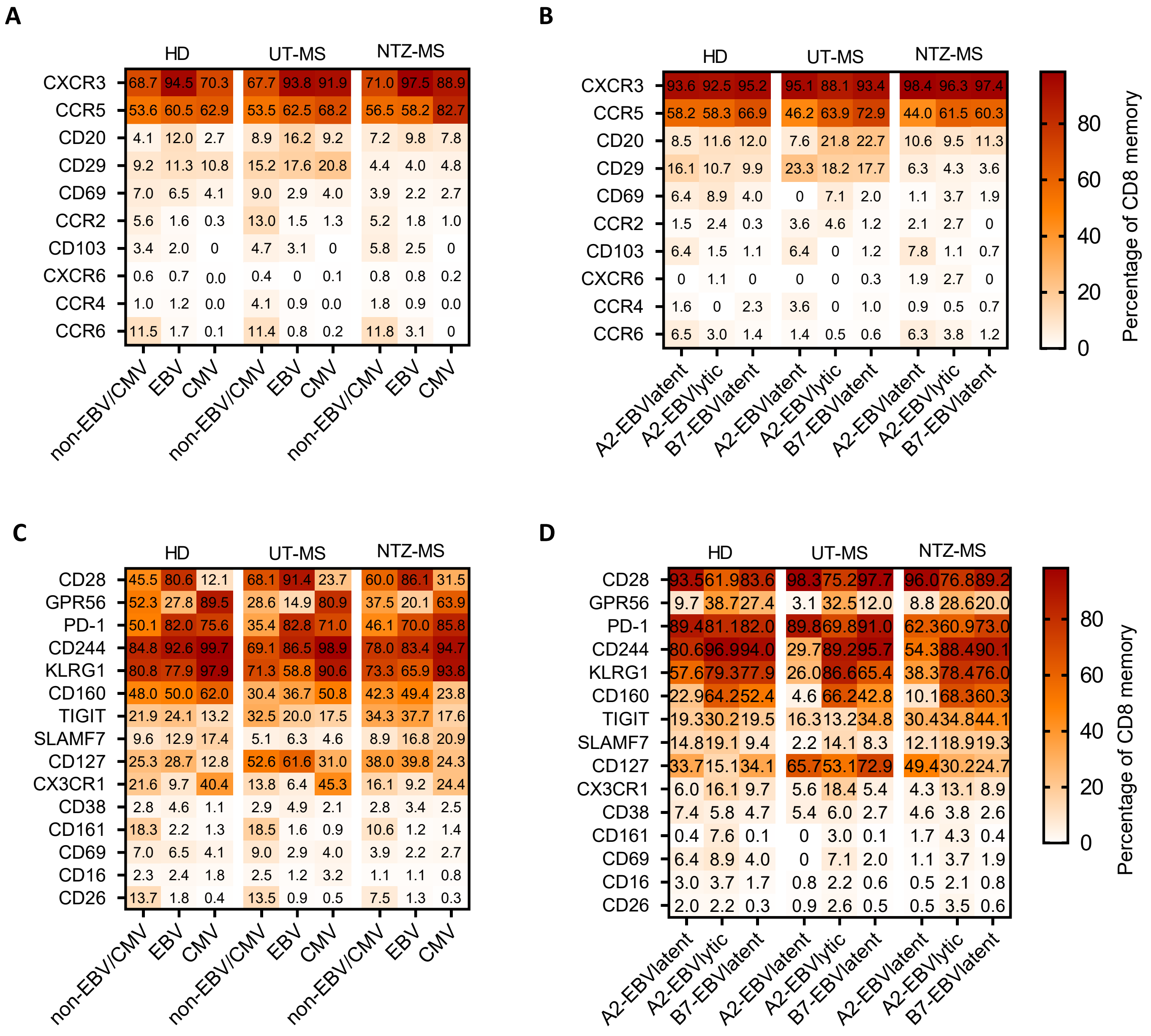

Figure S5. Heatmap of tissue-homing/residency associated markers and activation associated markers, related to Figure 3 and 4. Percentages of tissue-homing/residency associated markers for non-EBV/CMV CD8⁺ memory T cells, EBV- and CMV-specific CD8⁺ memory T cells **(A)** or for EBV- specific CD8⁺ memory T cells restricted to HLA-A2 or HLA-B7 with latent or lytic peptides **(B)**. Percentages of activation associated markers for non-EBV/CMV CD8⁺ memory T cells, EBV- and CMV-specific CD8⁺ memory T cells **(C)** or for EBV- specific CD8⁺ memory T cells restricted to HLA-A2 or HLA-B7 with latent or lytic peptides **(D)**.

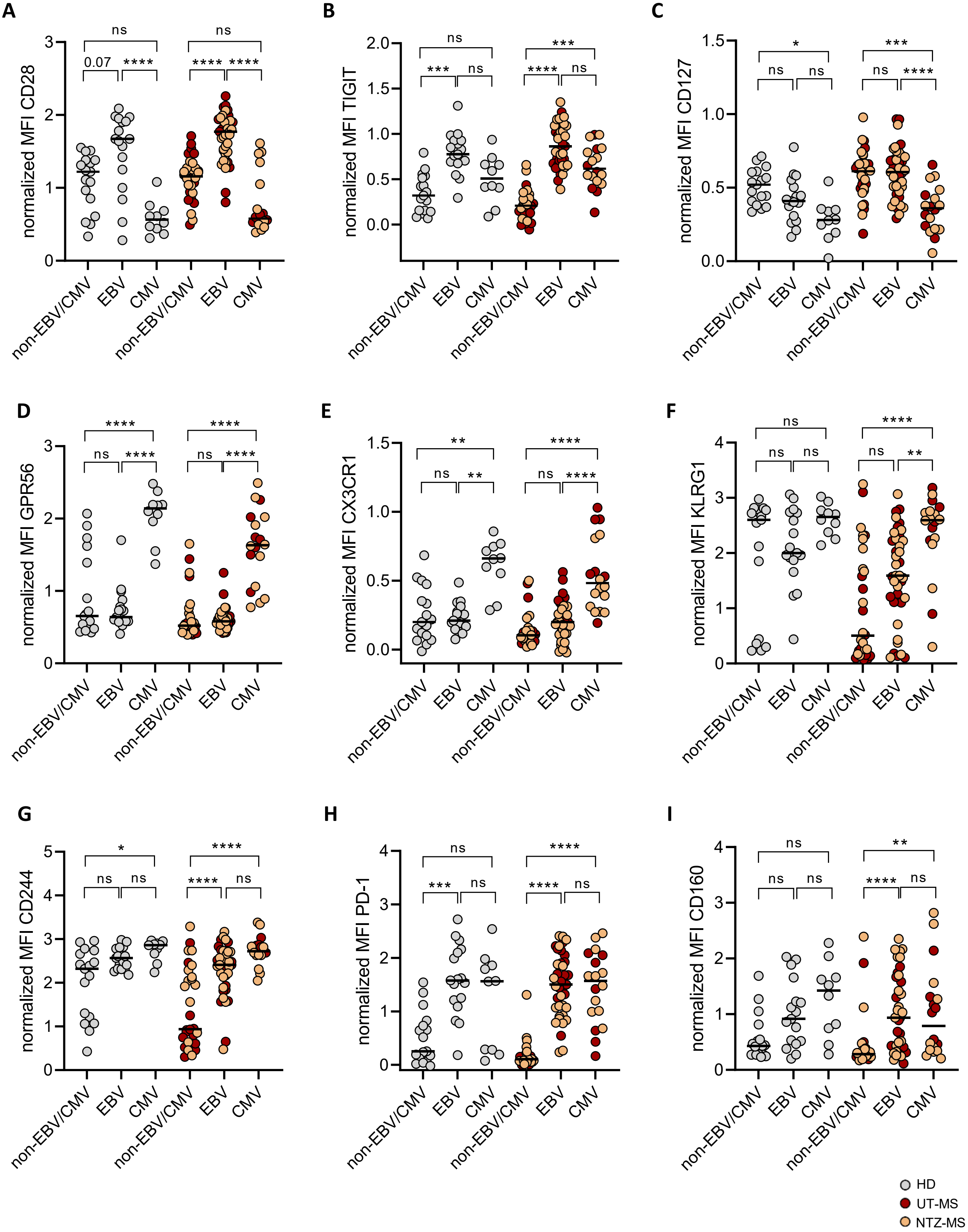

Figure S6. Differential marker expression between EBV- and CMV-specific CD8⁺ memory T cells, related to Figure 4. Normalized Median Fluorescence (MFI) of CD28 **(A)**, TIGIT **(B)**, CD127 **(C)**, GPR56 **(D)**, CX3CR1 **(E)**, KLRG1 **(F)**, CD244 **(G)**, PD-1 **(H)** and CD160 **(I)**. Kruskal-Wallis test with Dunn posthoc was used for statistical testing. Every dot represents one individual. Grey dots represent HD, red dots UT-MS and orange dots NTZ-MS. Lines represent medians. *p<0.05, **p<0.01, ***p<0.001, ****p<0.0001, ns = not significant.

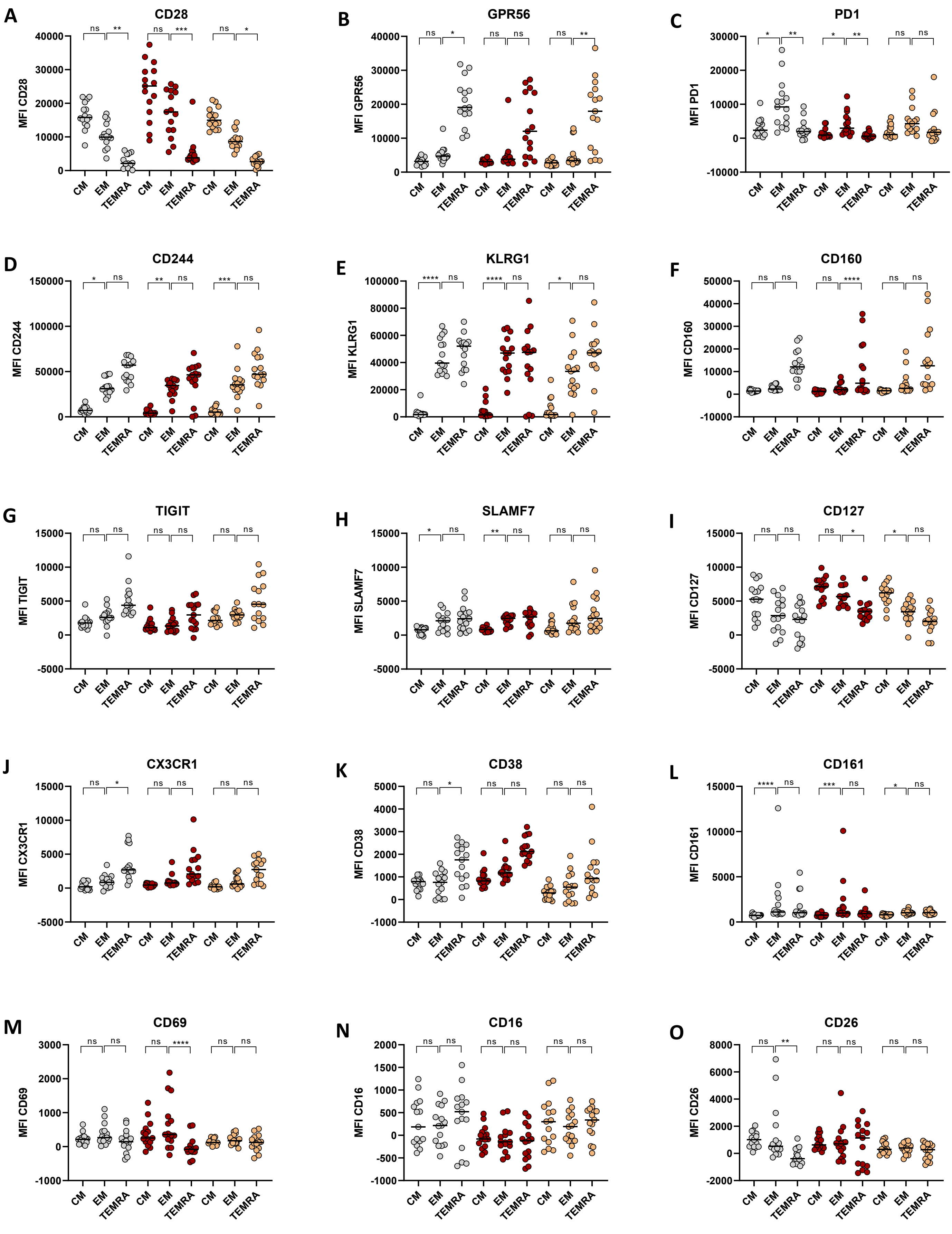

Figure S7. Differential marker expression between CD8$^+$ T_{CM}, T_{EM} and TEMRA cells for activation associated markers, related to Figure 4. Median Fluorescence (MFI) of CD28 **(A)**, GPR56 **(B)**, PD-1 **(C)**, CD244 **(D)**, KLRG1 **(E)**, CD160 **(F)**, TIGIT **(G)**, SLAMF7 **(H)**, CD127 **(I)**, CX3CR1 **(J)**, CD38 **(K)**, CD161 **(L)**, CD69 **(M)**, CD16 **(N)** and CD26 **(O)**. Kruskal-Wallis test with Dunn posthoc was used for statistical testing. Every dot represents one individual. Grey dots represent HD, red dots UT-MS and orange dots NTZ-MS. Lines represent medians. *p<0.05, **p<0.01, ***p<0.001, ****p<0.0001, ns = not significant.

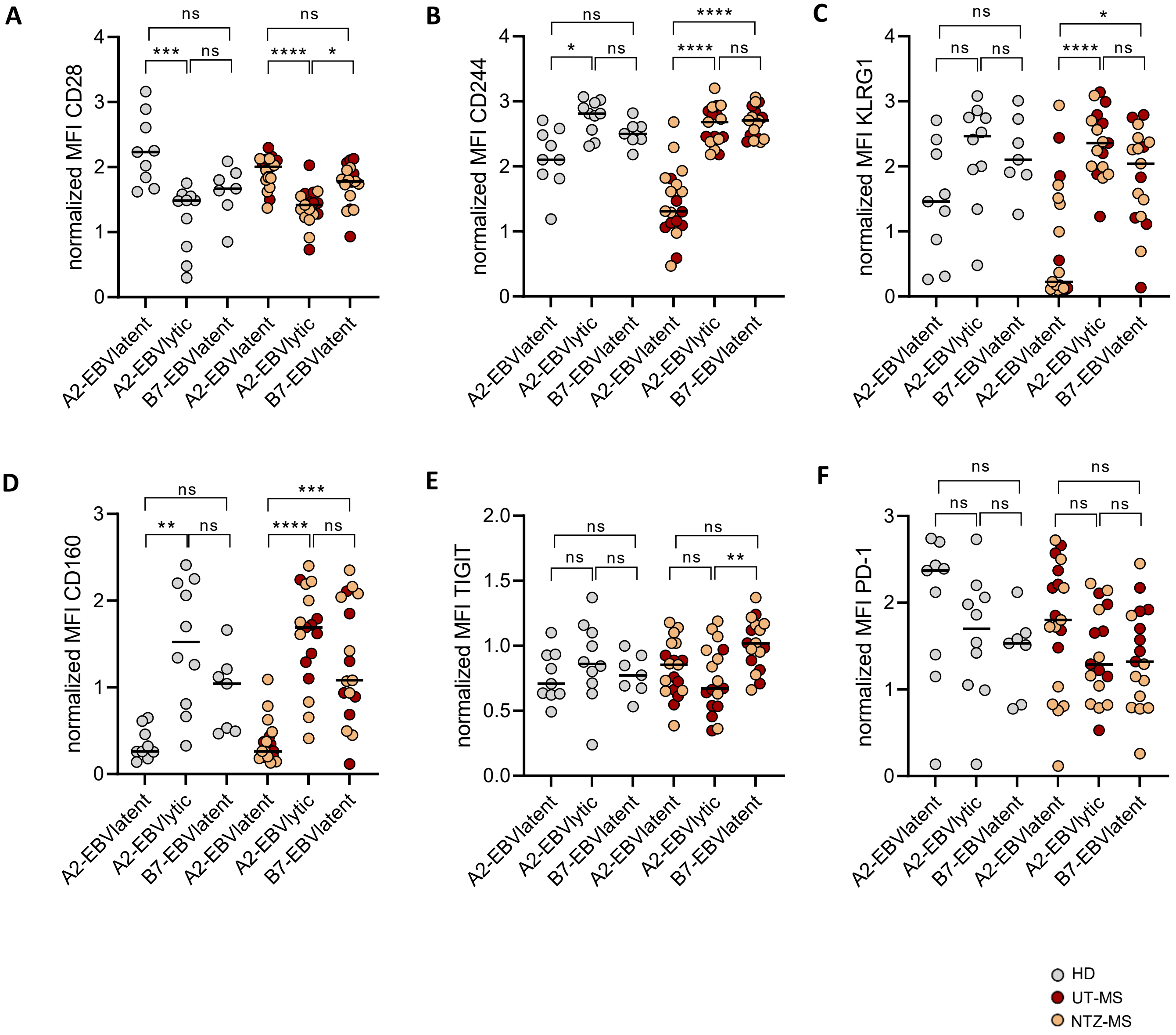

Figure S8. Co-stimulatory and co-inhibitory receptor expression on EBV-specific CD8⁺ memory T cells recognizing lytic or latent epitopes, related to Figure 4. Normalized Median Fluorescence (MFI) of CD28 **(A)**, CD244 **(B)**, KLRG1 **(C)**, CD160 **(D)**, TIGIT **(E)** and PD-1 **(F)**. Kruskal-Wallis test with Dunn posthoc was used for statistical testing. Every dot represents one individual. Grey dots represent HD, red dots UT-MS and orange dots NTZ-MS. Lines represent medians. *p<0.05, **p<0.01, ***p<0.001, ****p<0.0001, ns = not significant.

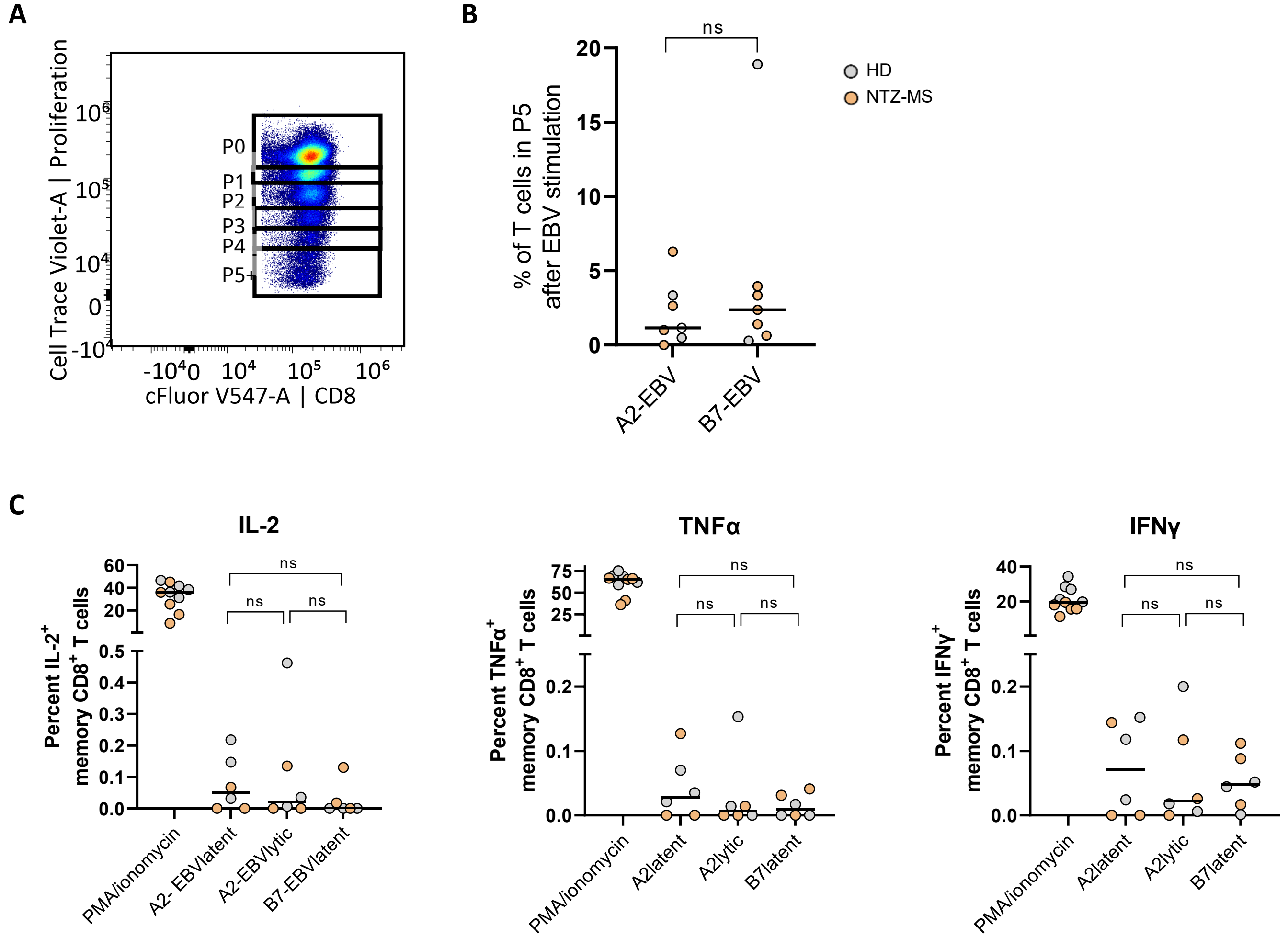

Figure S9. Proliferation and cytokine expression of EBV-specific CD8⁺ T cells after peptide stimulation, related to Figure 4. (A) Representative gating of 5 proliferation stadia of CD8⁺ T cells stained with CellTrace. **(B)** Percentage of cells in P5 after stimulation with HLA-A2 or HLA-B7 peptide pool. The percentage of cells in P5 with actin peptide pool stimulation is subtracted from the results. **(C)** Percentage of memory CD8⁺ T cells that produce IL-2, TNFa or IFNy 6 hours after stimulation with PMA/ionomycin, HLA-A2 EBVlatent peptide pool, HLA-A2 EBVlytic peptide pool or HLA-B7 EBVlatent peptide pool. The percentage of cells producing cytokines without stimulation is subtracted from the results. Grey dots represent HD and orange dots NTZ-MS. Lines represent medians. Mann-Whitney (B) and Kruskal-Wallis test with Dunn posthoc (C) were used for statistical testing. ns = not significant.

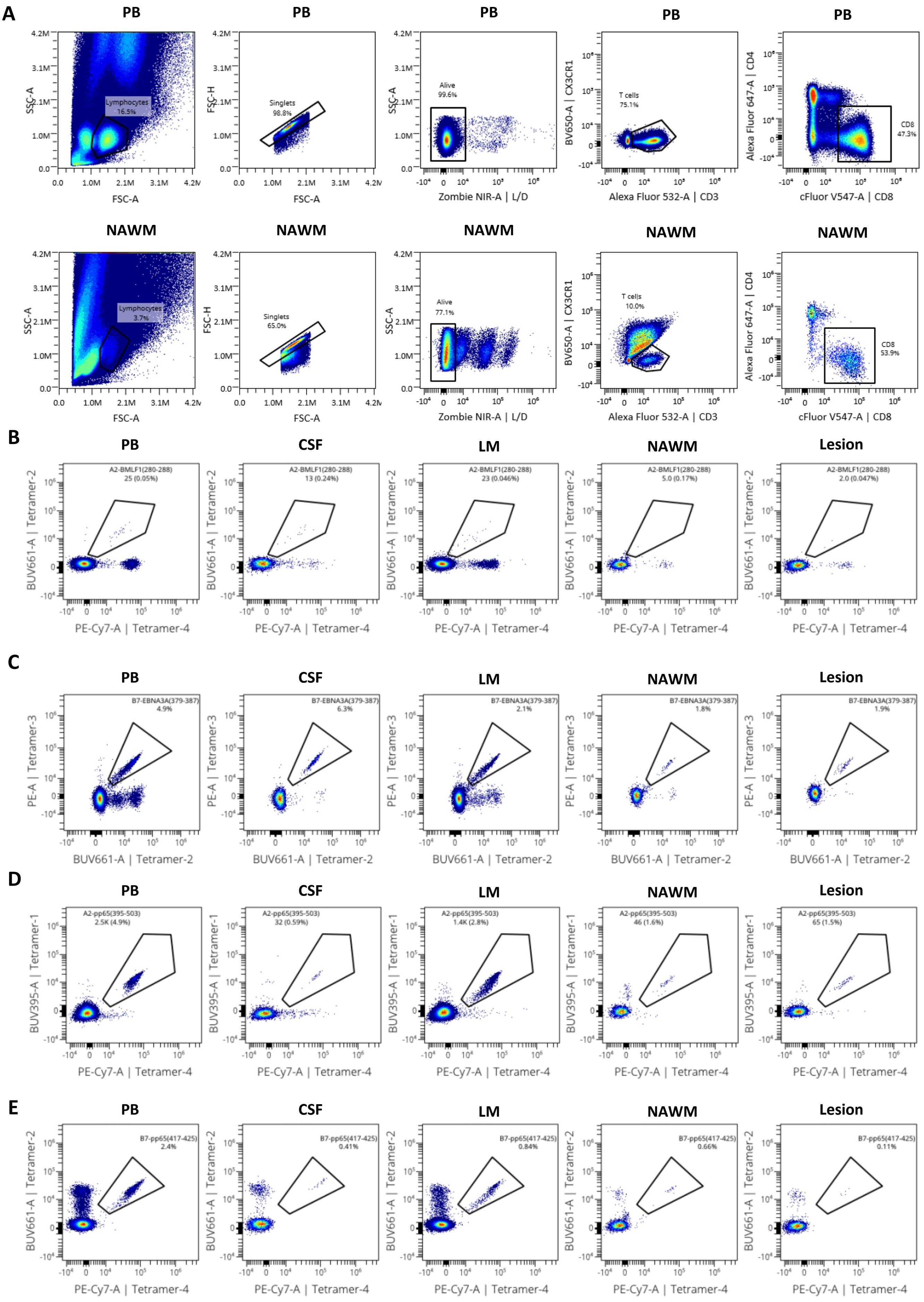

Figure S10. Gating of EBV- and CMV-specific CD8+ T cells in different post-mortem CNS compartments of an MS donor, related to Figure 5. Representative gating strategy of CD8+ T cells in PB and NAWM **(A)**, of EBV A2-BMLF1(280-288) **(B)**, of EBV B7-EBNA3A(379-387) **(C)**, of CMV A2-pp65(495-503) **(D)** and CMV B7-pp65(417-425) **(E)**. PB = peripheral blood, CSF = cerebrospinal fluid, LM = leptomeninges, NAWM = normal-appearing white matter.

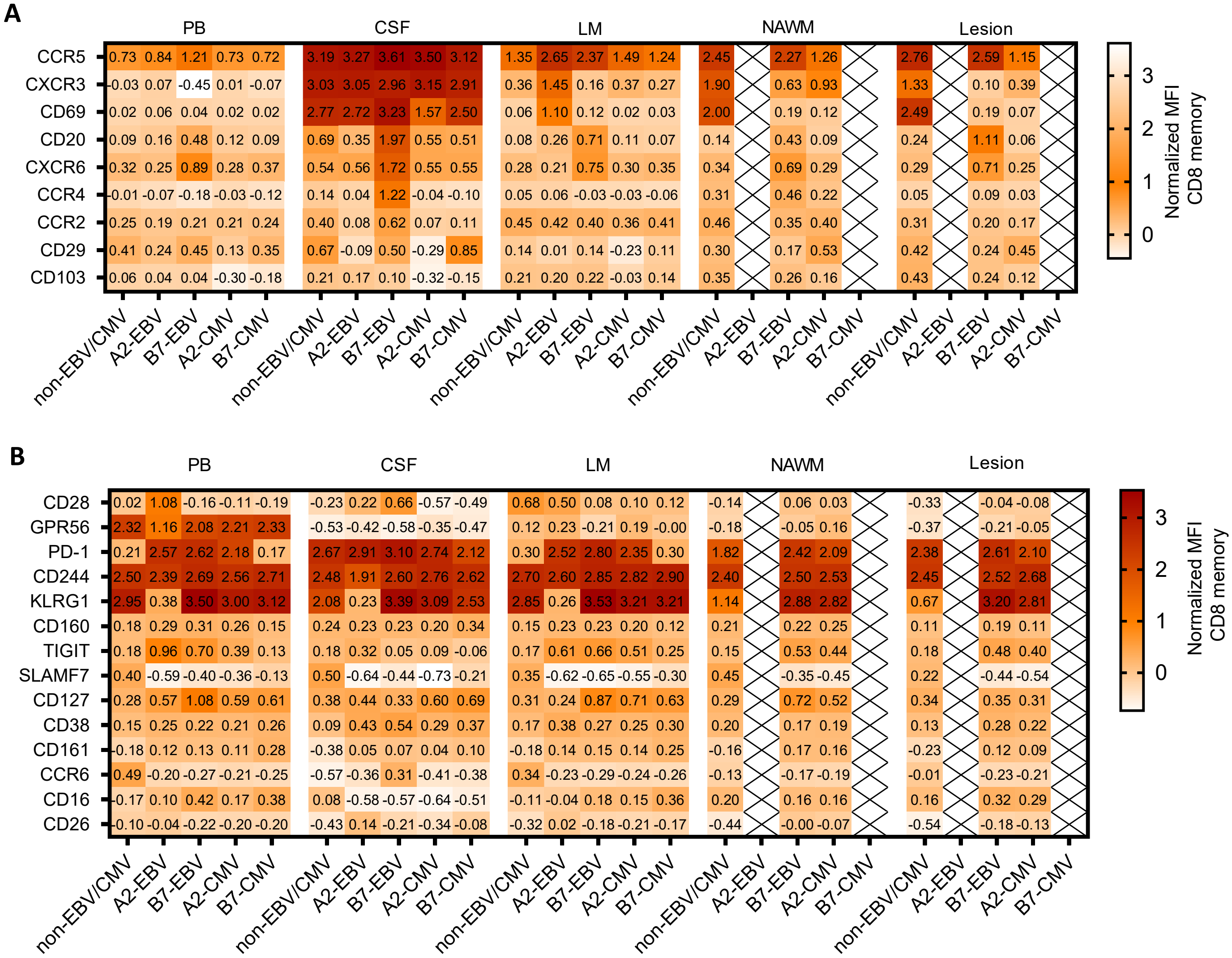

Figure S11. Tissue-homing/residency- and activation-associated markers in CNS compartments, related to Figure 5. Heatmap of median protein expression in normalized MFI for non-EBV/CMV CD8^{+} memory T cells, EBV- and CMV-specific CD8^{+} memory T cells for tissue-homing/residency-associated markers **(A)** and activation associated markers **(B)**.

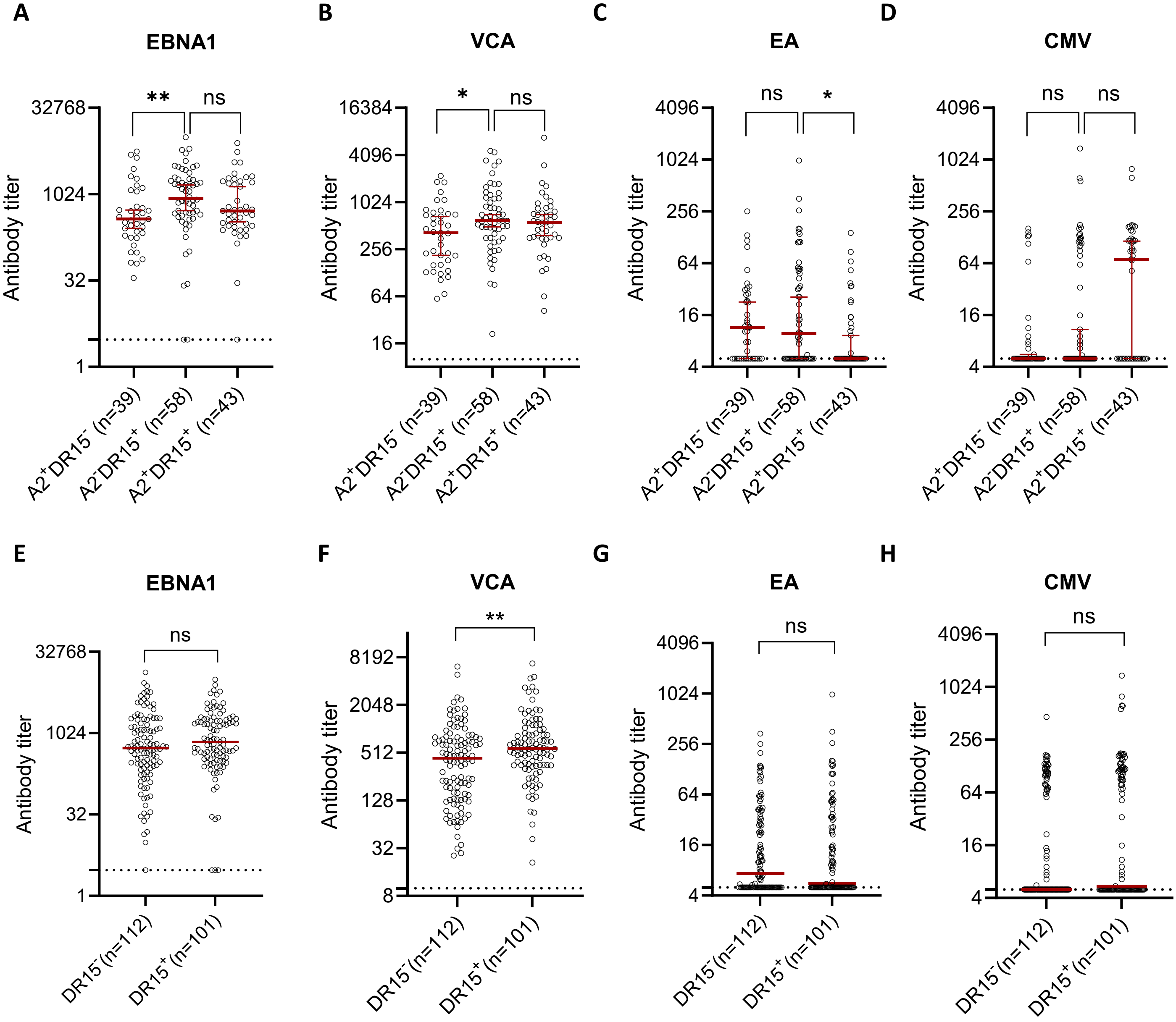

Figure S12. Antibody titers in pwMS stratified om HLA-DRB1*15:01, related to Figure 6. Antibody titers of HLA-A2 and HLA-DRB1*15:01 positive or negative persons with RRMS from the PROUD and MS-NTZ cohort. **(A)** EBNA1 **(B)** Early antigen (EA) **(C)** Viral capsid antigen (VCA) **(D)** CMV. Antibody titers of HLA-DRB1*15:01 positive or negative persons with RRMS from the PROUD and MS-NTZ cohort. **(E)** EBNA1 **(F)** Early antigen (EA) **(G)** Viral capsid antigen (VCA) **(H)** CMV. Kruskal-Wallis test with Dunn posthoc test (A-D) and Mann-Whitney test (E-H) were used for statistical testing. Error bars indicate 95% confidence intervals (A-D) and lines represent medians (E-H). *p<0.05, **p<0.01, ns = not significant.
